# Functional dependency level and later-life incident multimorbidity in older people: longitudinal evidence from 19 countries in China and Europe

**DOI:** 10.1186/s12889-025-25259-7

**Published:** 2025-12-24

**Authors:** Anying Bai, Yimin Qu, Qiushi Chen, Muir Gray, Simiao Chen, Yu Jiang

**Affiliations:** 1 School of Population Medicine and Public Health, Chinese Academy of Medical Sciences and Peking Union Medical College, Beijing, China; 2 Nuffield Department of Primary Care Health Sciences, University of Oxford, Oxford, UK; 3 School of Health Policy and Management, Chinese Academy of Medical Sciences and Peking Union Medical College, Beijing, China; 4 The Harold and Inge Marcus Department of Industrial and Manufacturing Engineering, The Pennsylvania State University, State College, PA, USA; 5 Heidelberg Institute of Global Health, Faculty of Medicine and University Hospital, Heidelberg University, Heidelberg, Germany

**Keywords:** Functional dependency, Older population, Multimorbidity, China, Europe

## Abstract

**Background:**

Population aging and increasing life expectancy raised concerns about functional dependency (FD) and multimorbidity. However, the impact of FD on later-life multimorbidity remains poorly understood.

**Method:**

Participants from the China Health and Retirement Longitudinal Study (CHARLS) and the Survey of Health, Ageing and Retirement in Europe (SHARE) with complete baseline FD and 7-year follow-up data on multimorbidity were included, excluding those with multimorbidity or missing specific chronic diseases at baseline. FD levels, measured by inability to perform basic activities of daily living (ADLs) and instrumental activities of daily living (IADLs) at baseline wave, were categorized into five cumulative-score groups. Multimorbidity was defined as the presence of two or more chronic diseases. Logistic regression was employed to analyze the association of FD with incident multimorbidity and individual chronic diseases in each cohort. Cohort-specific estimates were combined using random-effects meta-analysis. Stratified analyses and interaction tests assessed modifications of associations.

**Results:**

Compared to individuals without dependency, the risk of developing incident multimorbidity at 7-year follow-up with 2 FDs were significantly increased (2.13 [1.33–3.42] for ADL, 1.30 [1.02–1.66] for IADL), nearly doubling among patients with ≥ 4 FDs (1.52 [1.37–1.69] for ADL, 1.78 [1.18–2.69] for IADL). Significant associations between FDs and incident multimorbidity were observed across various subgroups, demonstrating dose-response relationships. Both cohorts exhibited positive interaction effects of age, gender, residential area, marital status, and social isolation on the associations between ADL dependency and incident multimorbidity.

**Conclusions:**

FD emerged as a significant risk factor for later-life multimorbidity, displaying interactions with demographic and social factors. This underscores the urgency for tailored interventions, integrated care models, and a reorientation of healthcare services to mitigate potential adverse health outcomes.

**Supplementary Information:**

The online version contains supplementary material available at 10.1186/s12889-025-25259-7.

## Background

 Facing rapid population aging [1] and prolonged life expectancy [2], many countries must confront the challenge of maintaining the functional status and quality of life of large numbers of older adults. With advancing age, some individuals may experience functional limitations, which in turn can lead to functional dependency (FD), a form of disability in which older adults require physical assistance due to degenerated body functions [3, 4]. The global prevalence of FD among the older adults was estimated to be approximately 39% in 54 countries in 2004 [5]. Previous research indicates that older adults in middle-income countries (MICs) are inadequately equipped to manage FD, with projections showing that the total number of Chinese older adults need care will increase from102.24 million in 2020 to 116.26 million by 2030 [6]. Difficulty in performing basic and instrumental activities of daily living associated with FD may increase the need for supportive care [7], contribute to higher medical expenditures [8], and negatively affect quality of life and health outcomes [9, 10]. 

As with FD, shifting age structures globally have raised concerns about the burden of chronic non-communicable diseases (NCDs). Multimorbidity, defined as the co-occurrence of two or more chronic diseases, becomes increasingly common with advancing age [11]. Globally, the pooled prevalence of multimorbidity reached 42.4% in 2020 [12], which has been estimated to affect up to 95% of individuals aged 65 years and older [13]. Patients with multimorbidity have higher probabilities of frailty [14], hospitalization [15], incurring catastrophic health expenditures [16], and mortality [17]. The impact of multimorbidity is intensified by declining physical function among older individuals and fragmented healthcare approaches [18]. Addressing individual diseases separately often leads to sub-optimal outcomes and complicates interactions with the healthcare system [19]. 

The increasing trends in FD and multimorbidity may have considerable financial implications over the next few decades [20]. In China, healthcare access disparities exist between urban and rural areas and among provinces, with financial barriers hindering care access, especially among the poor and those in less developed western provinces [21]. In addition, with current healthcare system focusing mainly on managing acute health conditions and chronic infectious diseases [22], China faces challenges related to a lack of disability-friendly living facilities and multimorbidity management. In Europe, despite mandates for universal health coverage, individuals facing socioeconomic challenges encounter difficulties in accessing care [23], although there are generally better provisions of living facilities and support for FD individuals [24]. Both China [25] and European [26] countries recognize the need for integrated care approaches to manage FD and multimorbidity, yet specific integrated care models are limited.

FD has typically been regarded as a nonfatal outcome of chronic conditions as people age in previous cross-sectional [27] and cohort studies [28, 29], but its predictive value for multimorbidity and associations with demographic and socioeconomic characteristics (SES) remain underexplored [30]. To address these gaps, we conducted a cross-cultural, longitudinal analysis based on two large prospective studies representing middle-aged and older adults across 19 countries: the China Health and Retirement Longitudinal Study (CHARLS) and the Survey of Health, Ageing and Retirement in Europe (SHARE). In this study, we hypothesized that higher levels of FD would be associated with an increased risk of incident multimorbidity and specific chronic diseases in both the CHARLS and SHARE cohorts. Furthermore, we anticipated that these associations would exhibit heterogeneity between the two cohorts, potentially due to differences in healthcare systems, socioeconomic contexts, and cultural factors between China and European countries. Unlike previous cross-sectional or single-country studies, our study provides longitudinal, cross-cultural evidence on the dose-response relationship between FD and incident multimorbidity. In addition, by incorporating mediation analysis, we extend prior work by examining the potential pathways through which FD influences the onset of multimorbidity, highlighting the role of mental–physical health comorbidity as potential mediators. We also assessed both cumulative FD and specific ADL/IADL impairments, stratified by demographic, socioeconomic, and psychosocial factors—an approach that has not been explored previously.

## Methods

### Study cohort

The seven-year longitudinal study utilized data from CHARLS [31] and SHARE [32], two large representative studies of middle-aged and older adults across 19 countries. CHARLS, initiated in 2011, included a baseline sample of 17,708 participants aged over 45 years from 450 villages or resident communities across 28 Chinese provinces, with follow-ups conducted every two years and new participants recruited in each subsequent wave. SHARE is a multidisciplinary and cross-national panel database from 18 European high-income countries (HICs). The Wave 4 of SHARE in 2011 consisted of approximately 65,000 individuals aged 50 or over. Among global aging studies, CHARLS and SHARE have recruited and followed up the largest numbers of participants from developing and developed countries, respectively. We used data from both CHARLS and SHARE to allow mutual validation and comparison of results between middle-income country (MIC) and HICs. We utilized two waves (2011 and 2018) of CHARLS and two waves (2011 and 2017) of SHARE to minimize temporal discrepancies. Information regarding study designs, participating countries, and specific waves was described extensively elsewhere [31, 32]. 

We included participants with complete data on FD and all covariates (as defined below) at baseline, as well as multimorbidity at follow-up. Individuals with missing information on any chronic conditions at either baseline or follow-up, or who had multimorbidity at or before baseline, were excluded. The detailed participant selection process for each cohort is provided in Supplement eMethods and illustrated in eFigure 2a. To enhance transparency regarding data availability, the extent of missingness for baseline covariates and follow-up chronic conditions is summarized in eTable 1.To address missing data, we generated ten imputed data sets using the multiple imputation method of covariates with missing values by chained equations (MICE) [33], and reperformed the main analyses as a sensitivity analysis to assess robustness.

### Follow-up of Multimorbidity and chronic diseases

Incident multimorbidity at follow-up was recorded as a dichotomous variable which was yes if the participants reported at least two of the chronic diseases [34]. In the CHARLS study, each participant’s disease status (yes or no) was collected in the baseline (2011) and follow-up (2018) survey waves for each of 14 NCDs including hypertension, asthma, arthritis, memory-related disease, psychiatric disease, digestive disease, kidney disease, stroke, heart disease, liver disease, chronic lung disease, cancer, diabetes, and dyslipidemia. In the SHARE study, each participant’s self-reported physician-diagnosed disease status (yes or no) was also collected in Wave 4 (2011) and follow-up (2017) for 15 NCDs, including hypertension, heart disease, high cholesterol, stroke, diabetes, lung disease, arthritis, osteoporosis, cancer or malignant tumor, digestive disease, Parkinson’s disease, cataracts, hip fracture, Alzheimer’s disease, and other types of fracture. The presence of these conditions was determined via self-reported history of a physician’s diagnosis obtained through an in-person visit with study personnel via a questionnaire. In the prospective analysis of an individual chronic disease, we have undertaken a reevaluation of the included cohort, concurrently excluding individuals with preexisting chronic condition at baseline in both cohorts.

### Definitions of functional dependency

Functional dependency (FD) means that people in older age require physical assistance due to degenerated body functions, placing substantial medical and care burdens on families and society [35]. In this study, FD is defined by a participant’s inability to perform basic activities of daily living (ADLs) and instrumental activities of daily living (IADLs). ADLs include dressing, bathing, eating, getting in/out bed, using the toilet, and controlling urination or defecation based on the Katz index of independence [36]. Controlling urination or defecation is not available for SHARE, and is replaced by walking across the room. IADLs include managing money, taking medication, shopping, meal preparation, doing housework and using the telephone based on the Lawton index of dependency [37]. Additionally, the ability to use a map to navigate is measured in SHARE. Both the ADL and IADL indices have a four-scale set of possible responses for each question: “no difficulty,” “have difficulty but can still do it,” “have difficulty and need help,” and “cannot do it.” In this study, responses to each question were dichotomized, with those who responded “have difficulty but can still do it,” “have difficulty and need help,” or “cannot do it” recorded as having difficulty. Cumulative FD level was then generated based on the number of ADLs and IADLs with reported difficulty, with scores ranging from 0 to 6 for ADLs in both CHARLS and SHARE, 0–6 for IADLs in CHARLS, and 0–7 for IADLs in SHARE. FD scores are categorized into four levels: no dependency, one dependency, two dependencies, three dependencies, and ≥ 4 dependencies, with higher values indicating greater FD severity.

### Covariates

Covariates were identified by use of an a priori theory-driven approach. Data on demographics (age and gender), socio-economic characteristics (education, living region, and household wealth level), living arrangements (marital status), lifestyles (smoking and body mass index), mental symptoms (depressive symptoms), and social isolation were collected. More details about other variables are provided in the eMethods in the Supplement.

### Statistical analysis

Baseline participant characteristics were summarized by ADL and IADL dependency status. Continuous variables were reported as means ± standard deviations (SDs), and categorical variables as frequencies with percentages. Differences in characteristics were assessed using the t test, Wilcoxon rank-sum test, or χ^2^ test as appropriate.

For each cohort, multivariable logistic models were used to investigate the associations between FD level and each individual component of FD with incident multimorbidity, adjusted for covariates of age, gender, marital status, educational level, residential area, smoking, household wealth, depressive symptoms, body mass index (BMI) category and social isolation assessed at baseline. Stratified and interaction analyses by covariates were further conducted to explore heterogeneity in FD’s effect, except for depressive symptoms in CHARLS and household wealth levels in SHARE due to sample size limitations. Trend tests were performed to assess dose-response associations overall and within subgroups [38]. Additionally, a meta-analysis was conducted to estimate the pooled effect size of FD levels on incident multimorbidity across both cohorts, following Cochrane Handbook guidelines [39]. Adjusted odds ratios (AORs) and 95% CIs were reported for all regression models.

We further explored the association between FD level and incidence of each individual chronic disease using the multivariable logistic models adjusted for the same set of covariates listed above. Trend tests were also performed to evaluate dose-response associations. All statistical analyses were conducted using STATA software (version 16.0; Stata Corp LP, College Station, TX, USA).

## Results

**Fig. 1:**
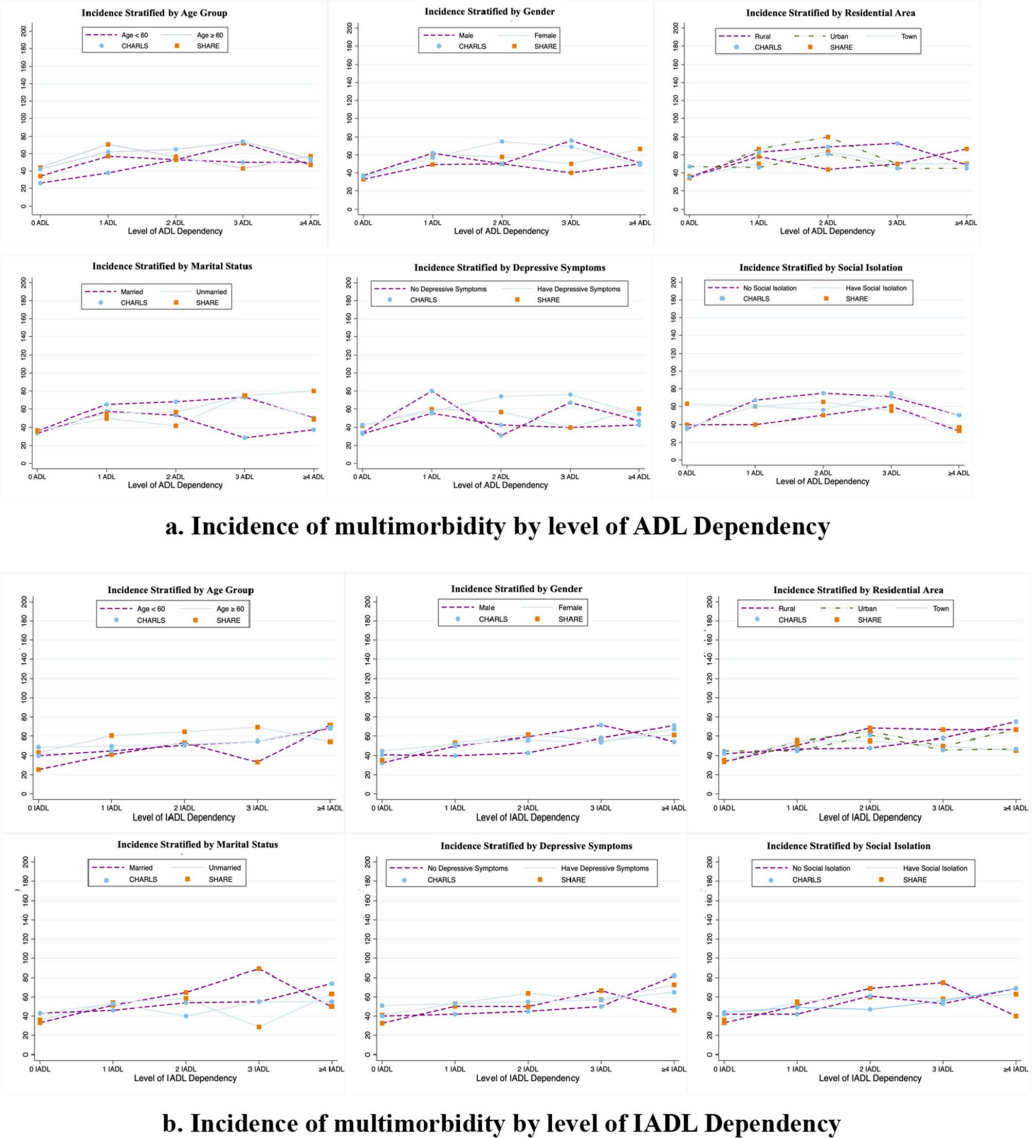
Incidence of follow-up multimorbidity by level of functional dependency in two cohorts stratified by demographic characteristics, socioeconomic status, mental health and social isolation. **a** Incidence of multimorbidity by level of ADL Dependency.** b** Incidence of multimorbidity by level of IADL Dependency

**Fig. 2:**
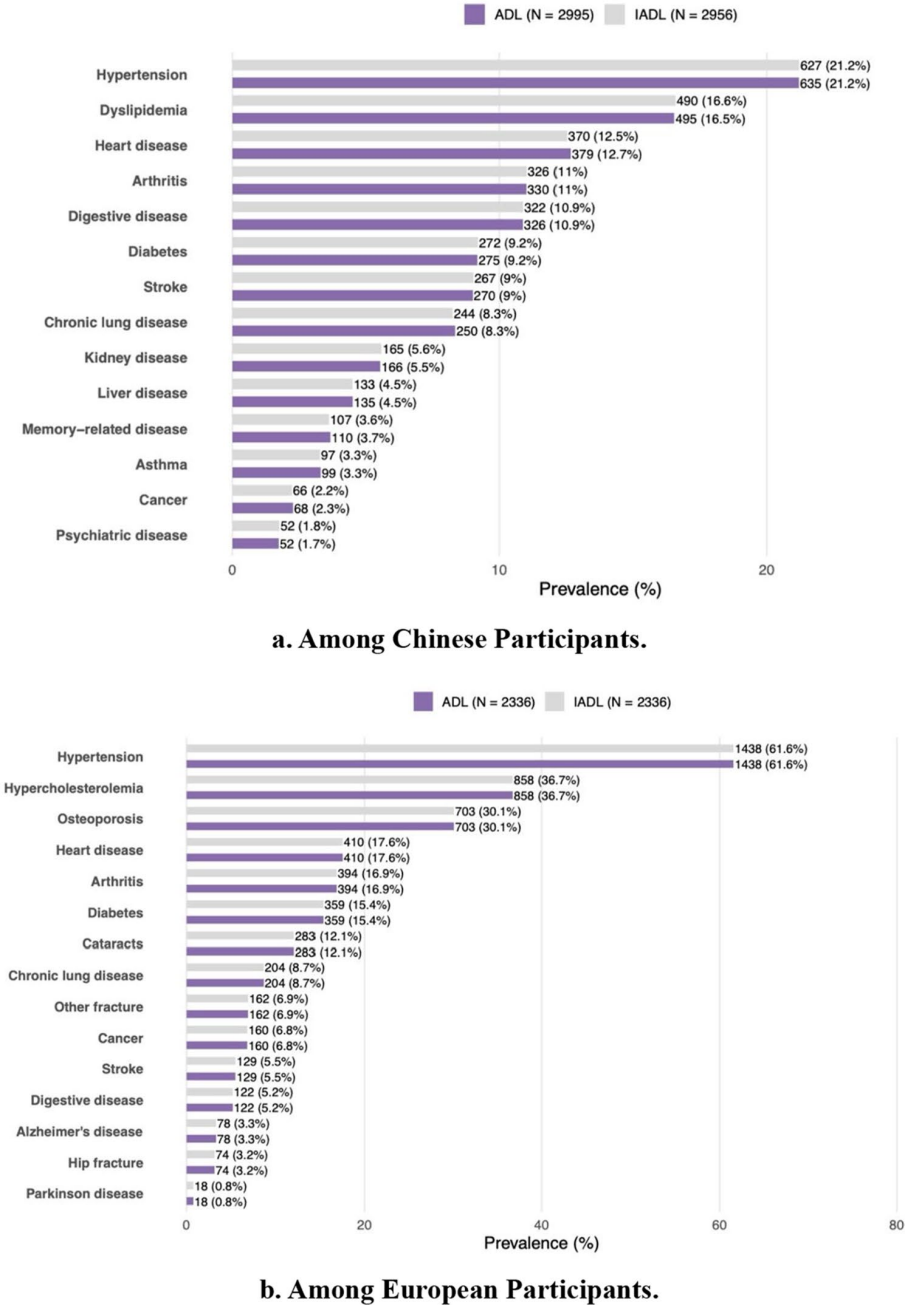
Prevalence of Chronic Diseases among Chinese and European Participants with Incident Multimorbidity. ** a **Among Chinese Participants. **b** Among European Participants

Following the cohort selection criteria, we included 6,773 participants for ADL dependency analysis and 6,692 for IADL dependency from CHARLS, and 6,574 participants for both analysis from SHARE. Participants with FD were more likely to be older, less educated, rural residence, underweight, current smokers, with lower household wealth, and experiencing depressive symptoms and social isolation, with elevated risks of incident multimorbidity or chronic diseases in both cohorts (eTables 2 and 3). Multimorbidity incidence was higher in China compared to Europe, with elevated rates among older, rural-dwelling, depressed, and socially isolated participants in both cohorts (Fig. 1). We further analyzed the spectrum of chronic diseases among participants with incident multimorbidity. In CHARLS, the three most common chronic conditions were hypertension (21.2%), dyslipidemia (16.6%), and heart disease (12.7%), with higher prevalence of digestive disease (11%), diabetes (9.2%), and stroke (9%). In SHARE, hypertension (61.6%), hypercholesterolemia (36.7%), and osteoporosis (30.1%) were most prevalent, followed by heart disease (17.6%), arthritis (16.9%) and diabetes (15.4%). Despite some notable differences, both cohorts shared several patterns: hypertension, dyslipidemia/hypercholesterolemia, and heart disease were consistently among the most prevalent chronic conditions (Fig. 2).

In CHARLS, participants with ≥4 FDs had increased odds of multimorbidity (ADL: 1.52 [1.37 - 1.69]; IADL: 1.96 [1.21 – 3.17]). For those with 2 ADLs, the odds ratio was 2.06 (95% CI 1.08 - 3.95) (Table 1). In SHARE, participants with 2 FDs had more than double the risk (ADL: 2.21 [1.11 – 4.44]; IADL: 2.27 [1.42 – 3.64]), while those with 1 FD had nearly twice the risk of incident multimorbidity (1.92 [1.39 – 2.66] for ADL, 1.66 [1.31 – 2.11] for IADL) (Table 2). Additionally, significant dose-response associations were found between FD levels and incident multimorbidity in both cohorts. Bathing, housekeeping, and shopping independently increased incident multimorbidity risk in both cohorts. Remarkably, requiring assistance with bathing and toileting exhibited substantial associations, leading to an increased risk of developing multimorbidity by more than twofold in SHARE and almost twofold in CHARLS (eFigure 2). Meta-analysis confirmed significant associations between FDs and incident multimorbidity (2.13 [1.33-3.42] for 2 ADLs and 1.30 [1.02 - 1.66] for 2 IADLs, 1.52 [1.37 -1.69] for ≥4 ADLs and 1.78 [1.18-2.69] for ≥4 IADLs) (eFigure 3).

**Table 1:** Association between functional dependency level and incident Multimorbidity in the overall cohort and subgroups of CHARLS

	ADL^a^						IADL^b^					
	1^c^	2	3	≥ 4	P value	P value	1^c^	2	3	≥ 4	P value	P value
					for trend	for interaction in ADL					for trend	for interaction in IADL
Overall	1.66 (0.76–3.61)	2.06 (1.08–3.95)	3.70 (1.98–6.93)	1.52 (1.37–1.69)	< 0.001		1.05 (0.88–1.27)	1.07 (0.81–1.42)	1.22 (0.82–1.81)	1.96 (1.21–3.17)	0.012	
Subgroup												
Age												
< 60	1.48 (0.32–6.77)	2.03 (0.77–5.37)	4.33 (1.51–12.37)	1.58 (1.39–1.81)	< 0.001	< 0.001	1.13 (0.88–1.45)	1.27 (0.85–1.91)	1.59 (0.84–3.02)	2.25 (0.99–5.09)	0.007	0.005
≥ 60	1.76 (0.71–4.39)	2.24 (0.93–5.42)	3.70 (1.69–8.12)	1.49 (1.24–1.78)	< 0.001	< 0.001	0.97 (0.74–1.27)	0.99 (0.67–1.45)	1.08 (0.66–1.77)	1.93 (1.07–3.49)	0.149	0.005
Gender												
Male	1.35 (0.29–6.21)	4.42 (1.40–14.00)	3.53 (1.21–10.34)	1.58 (1.36–1.84)	< 0.001	< 0.001	0.89 (0.67–1.19)	0.91 (0.57–1.45)	1.46 (0.71–2.98)	2.65 (1.14–6.16)	0.165	0.006
Female	1.69 (0.68–4.20)	1.27 (0.55–2.91)	3.52 (1.61–7.69)	1.46 (1.26–1.70)	< 0.001	< 0.001	1.16 (0.91–1.48)	1.18 (0.83–1.69)	1.11 (0.69–1.78)	1.67 (0.93–3.01)	0.053	0.006
Residence Area												
Urban	1.06 (0.14–7.84)	0.78 (0.22–2.78)	3.01 (0.78–11.56)	1.62 (1.35–1.93)	< 0.001	< 0.001	0.88 (0.61–1.27)	1.33 (0.76–2.33)	0.65 (0.28–1.52)	0.66 (0.26–1.69)	0.456	0.991
Rural	1.74 (0.74–4.09)	3.15 (1.40–7.07)	3.87 (1.90–7.87)	1.48 (1.29–1.69)	< 0.001	< 0.001	1.12 (0.91–1.39)	1.01 (0.73–1.40)	1.46 (0.93–2.29)	2.89 (1.61–5.18)	0.001	0.991
Marital status												
Married	2.07 (0.81–5.30)	2.78 (1.24–6.28)	3.62 (1.78–7.34)	1.53 (1.36–1.71)	< 0.001	< 0.001	1.02 (0.83–1.25)	1.24 (0.90–1.69)	1.20 (0.77–1.86)	2.55 (1.42–4.59)	0.004	0.124
Bachelordom	1.02 (0.24–4.43)	1.05 (0.31–3.49)	4.61 (1.17–18.14)	1.49 (1.11–1.99)	< 0.001	< 0.001	1.25 (0.81–1.94)	0.65 (0.33–1.26)	1.29 (0.53–3.13)	1.10 (0.45–2.69)	0.894	0.124
Depressive Symptoms												
No	6.06 (0.66–56.16)	0.69 (0.21–2.31)	3.34 (0.98–11.36)	1.56 (1.37–1.77)	< 0.001	< 0.001	1.03 (0.80–1.32)	1.11 (0.70–1.74)	1.28 (0.64–2.55)	6.21 (1.75–22.02)	0.029	< 0.001
Yes	1.28 (0.54–3.04)	3.59 (1.48–8.75)	3.83 (1.82–8.07)	1.44 (1.18–1.76)	< 0.001	< 0.001	1.08 (0.82–1.42)	1.06 (0.74–1.52)	1.21 (0.75–1.96)	1.52 (0.89–2.57)	0.11	< 0.001
Household Wealth Level												
Low	11.32 (1.38–93.14)	1.62 (0.58–4.53)	5.57 (2.01–15.44)	1.56 (1.28–1.89)	< 0.001	< 0.001	0.99 (0.71–1.38)	1.17 (0.73–1.88)	1.12 (0.60–2.10)	2.65 (1.15–6.09)	0.062	0.023
Medium	0.82 (0.27–2.51)	2.59 (0.78–8.64)	4.12 (1.30–13.10)	1.37 (1.14–1.66)	< 0.001	< 0.001	0.92 (0.67–1.27)	1.09 (0.65–1.84)	1.31 (0.59–2.92)	1.28 (0.58–2.81)	0.511	0.023
High	0.82 (0.15–4.39)	2.08 (0.61–7.07)	1.85 (0.56–6.12)	1.63 (1.37–1.94)	< 0.001	< 0.001	1.27 (0.93–1.73)	0.99(0.61–1.60)	1.32 (0.68–2.56)	2.26 (0.90–5.72)	0.067	0.023
Social Isolation												
No	3.01 (0.26–34.97)	4.48 (0.88–22.95)	3.15 (1.07–9.25)	1.64 (1.39–1.93)	< 0.001	< 0.001	0.88 (0.63–1.23)	1.75 (1.05–2.91)	1.12 (0.54–2.32)	2.18 (0.73–6.51)	0.089	0.039
Yes	1.57 (0.69–3.58)	1.75 (0.85–3.59)	4.04 (1.86–8.77)	1.45 (1.26–1.66)	< 0.001	< 0.001	1.14 (0.91–1.42)	0.88 (0.62–1.23)	1.27 (0.80–2.03)	1.98 (1.16–3.37)	0.041	0.039

^a^ADL: Activity of Daily Living.^d^Odds Ratio (95% CI). Models in overall population were fully adjusted for age, gender, marital status, educational level, rural or urban residence, smoking status, household wealth level, depressive symptoms, social isolation, and body mass index (BMI) category. In subgroup analysis, all other covariates were adjusted for except the stratified variable.

^b^IADL: Instrumental Activity of Daily Living

^c^Reference: No Functional Dependency

^d^Odds Ratio (95% CI). Models in overall population were fully adjusted for age, gender, marital status, educational level, rural or urban residence, smoking status, household wealth level, depressive symptoms, social isolation, and body mass index (BMI) category. In subgroup analysis, all other covariates were adjusted for except the stratified variable

**Table 2:** Association between functional dependency level and incident Multimorbidity in the overall cohort and subgroups of SHARE

	ADL^a^						IADL^b^					
	1^c^	2	3	≥ 4	P value	P value	1	2	3	≥ 4	P value	P value
					for trend	for interaction in ADL					for trend	for interaction in IADL
Overall	1.92 (1.39–2.66)^d^	2.21 (1.11–4.44)	1.01 (0.25–4.05)	1.77 (0.56–5.63)	< 0.001	< 0.001	1.66 (1.31–2.11)	2.27 (1.42–3.64)	1.5 (0.53–4.27)	1.35 (0.60–3.03)	< 0.001	< 0.001
Subgroup												
Age												
< 60	1.48 (0.90–2.45)	3.09 (1.17–8.20)	2.68 (0.37–19.62)	1.88 (0.37–9.58)	0.009	< 0.001	1.83 (1.25–2.68)	2.81 (1.30–6.09)	3.05 (0.19–49.14)	5.72 (1.09–30.11)	< 0.001	< 0.001
≥ 60	2.55 (1.64–3.98)	1.80 (0.71–4.58)	0.61 (0.10–3.78)	1.58 (0.31–7.94)	0.004	< 0.001	1.80 (1.33–2.45)	2.31 (1.30–4.12)	2.03 (0.67–6.17)	1.02 (0.42–2.51)	< 0.001	< 0.001
Gender												
Male	1.50 (0.89–2.51)	2.21 (0.71–6.90)	0.65 (0.06–6.72)	2.14 (0.61–7.52)	0.052	< 0.001	1.68 (1.05–2.68)	2.70 (1.06–6.87)	2.68 (0.50–14.31.50.31)	1.78 (0.48–6.58)	0.004	< 0.001
Female	2.21 (1.45–3.38)	2.15 (0.89–5.19)	1.31 (0.21–8.34)	1.08 (0.07–17.53)	0.001	< 0.001	1.63 (1.23–2.16)	2.16 (1.24–3.74)	1.01 (0.26–3.94)	1.12 (0.41–3.10)	0.001	< 0.001
Residence Area												
Urban	2.88 (1.29–6.42)	6.59 (1.33–32.74)	2.01 (0.12–33.03)	0.82 (0.07–9.86)	0.013	< 0.001	1.55 (1.08–2.23)	1.67 (0.91–3.04)	0.34 (0.06–1.79)	1.48 (0.53–4.17)	0.024	< 0.001
Town	1.38 (0.78–2.43)	2.29 (0.63–8.30)	1.30 (0.15–10.90)	1.92 (0.37–9.99)	0.097	< 0.001	1.75 (1.27–2.40)	3.62 (1.67–7.86)	8.04 (0.96–67.02)	1.22 (0.33–4.52)	0.065	< 0.001
Rural	2.12 (1.34–3.37)	1.20 (0.41–3.53)	0.45 (0.04–5.63)	4.42 (0.39–49.77)	0.014	< 0.001	1.69 (0.98–2.90)	2.31 (0.81–6.62)	0.88 (0.05–14.52)	2.44 (0.36–16.58)	0.001	< 0.001
Marital status												
Married	2.19 (1.42–3.39)	2.74 (0.98–7.65)	0.72 (0.12–4.21)	1.00 (0.23–4.31)	0.014	< 0.001	1.53 (1.07–2.19)	3.54 (1.71–7.33)	1.55 (0.45–5.38)	1.58 (0.43–5.85)	< 0.001	< 0.001
Bachelordom	1.63 (1.00–2.64.00.64)	1.84 (0.73–4.68)	1.94 (0.15–24.93)	5.72 (0.58–56.97)	0.007	< 0.001	1.83 (1.22–2.74)	1.38 (0.62–3.05)	0.97 (0.06–17.16)	1.05 (0.30–3.72)	< 0.001	< 0.001
Social Isolation												
No	2.62 (1.60–4.28)	1.87 (0.64–5.42)	1.16 (0.19–7.06)	1.06 (0.17–6.49)	0.009	0.004	1.88 (1.33–2.65)	4.19 (1.79–9.78)	6.10 (0.63–59.08)	1.02 (0.17–6.19)	< 0.001	0.004
Yes	1.64 (1.09–2.48)	2.42 (1.00–5.85.00.85)	1.35 (0.17–10.60)	2.24 (0.48–10.40)	0.004	0.004	1.84 (1.34–2.53)	2.08 (1.20–3.61)	1.88 (0.59–6.03)	1.82 (0.76–4.36)	< 0.001	0.004

^a^ADL Activity of Daily Living

^b^IADL: Instrumental Activity of Daily Living

^c^Reference: No Functional Dependency

^d^Odds Ratio (95% CI). Models in overall population were fully adjusted for age, gender, marital status, educational level, rural or urban residence, smoking status, household wealth level, depressive symptoms, social isolation, and body mass index (BMI) category. In subgroup analysis, all other covariates were adjusted for except the stratified variable

Significant associations of FD and multimorbidity were also found in subgroups with significant dose-response associations (Tables 1 and 2). Subgroup analysis revealed stronger associations among older individuals, rural residents, and those with depressive symptoms and lower wealth in CHARLS, which, however, were not observed in SHARE. Both cohorts showed positive modification effect of age, gender, residential area, marital status, and social isolation on the associations between ADL dependency and incident multimorbidity, which underscores the significance of taking into account these factors when managing and preventing multimorbidity among older adults. Results obtained with the imputed data sets were consistent with those from the main analyses, indicating the robustness of these findings (eTable 4).

In the analysis of associations between FD level and incident individual chronic disease, we observed significant dose-response associations between cumulative FD level and incident heart disease, arthritis, stroke, psychiatric disease (Parkinson disease in SHARE) and memory-related disease (Alzheimer’s disease in SHARE) in both cohorts. Significant dose-response associations were observed between FD levels and incident dyslipidemia and cancer among CHARLS cohort. Similarly, dose-response associations were observed between FD levels and incident hypercholesterolemia, chronic lung disease, hip fracture, other fracture, cataracts, osteoporosis, and digestive disease among SHARE cohort (Tables 3 and 4).

**Table 3:** Association between functional dependency level and incident chronic diseases in CHARLS

	Adjusted odds ratio ^a^ (95%CI) by Functional Dependency Level					
Chronic disease	0	1	2	3	≥ 4	P-value for trend
ADL^b^ Dependency						
Hypertension (*N* = 7,098)	1[Reference]	1.00 (0.45–2.22)	0.93 (0.47–1.82)	1.51 (0.89–2.55)	1.07 (0.92–1.24)	0.401
Dyslipidemia (*N* = 8,846)	1[Reference]	1.65 (0.79–3.45)	1.35 (0.70–2.61)	0.64 (0.31–1.34)	1.32 (1.13–1.55)	0.001
Diabetes (*N* = 9,606)	1[Reference]	0.63 (0.19–2.07)	1.21 (0.54–2.71)	0.99 (0.48–2.03)	1.21 (0.99–1.49)	0.051
Heart disease (*N* = 8,816)	1[Reference]	0.99 (0.35–2.81)	1.35 (0.60–3.02)	2.11 (1.19–3.73)	1.58 (1.30–1.91)	< 0.001
Stroke (*N* = 10,364)	1[Reference]	2.05 (0.89–4.69)	2.00 (1.03–3.90)	1.47 (0.81–2.67)	1.23 (1.01–1.50)	0.083
Chronic lung disease (*N* = 9,219)	1[Reference]	1.35 (0.52–3.48)	2.48 (1.23–5.01)	1.47 (0.76–2.84)	1.20 (0.97–1.49)	0.151
Asthma (*N* = 10,073)	1[Reference]	2.38 (0.80–7.05)	0.88 (0.21–3.73)	1.34 (0.51–3.51)	1.31 (0.93–1.85)	0.163
Liver disease (*N* = 10, 025)	1[Reference]	0.84 (0.20–3.53)	2.41 (1.07–5.46)	0.60 (0.19–1.96)	1.14 (0.88–1.49)	0.355
Cancer (*N* = 10, 541)	1[Reference]	2.63 (0.60–11.58.60.58)	0.90 (0.12–6.78)	0.99 (0.23–4.29)	1.28 (0.85–1.93)	0.283
Digestive disease (*N* = 7, 340)	1[Reference]	0.47 (0.11–1.97)	1.44 (0.60–3.43)	0.85 (0.38–1.89)	1.19 (0.98–1.45)	0.06
Kidney disease (*N* = 9,654)	1[Reference]	0.78 (0.19–3.27)	1.68 (0.65–4.30)	2.07 (1.06–4.06)	1.26 (0.98–1.62)	0.083
Arthritis (*N* = 5,804)	1[Reference]	0.84 (0.19–3.64)	1.72 (0.58–5.04)	1.61 (0.66–3.94)	1.26 (1.03–1.53)	0.025
Psychiatric disease (*N* = 10,500)	1[Reference]	2.75 (0.61–12.43)	1.01 (0.13–7.72)	3.37 (1.27–8.95)	1.87 (1.13–3.12)	0.024
Memory-related disease (*N* = 10,458)	1[Reference]	2.73 (0.91–8.16)	3.29 (1.32–8.20)	3.80 (1.84–7.81)	2.07 (1.45–2.95)	< 0.001
IADL^c^ Dependency						
Hypertension (*N* = 7, 079)	1[Reference]	1.17 (0.93–1.46)	0.81 (0.57–1.13)	0.89 (0.57–1.39)	1.18 (0.76–1.83)	0.984
Dyslipidemia (*N* = 8, 816)	1[Reference]	0.89 (0.69–1.15)	1.06 (0.76–1.47)	1.37 (0.91–2.05)	1.27 (0.83–1.96)	0.172
Diabetes (*N* = 9,574)	1[Reference]	1.21 (0.92–1.61)	0.99 (0.65–1.51)	1.44 (0.89–2.33)	0.86 (0.49–1.49)	0.593
Heart disease (*N* = 8,785)	1[Reference]	1.07 (0.82–1.41)	1.20 (0.83–1.73)	1.88 (1.26–2.80)	1.15 (0.69–1.92)	0.02
Stroke (*N* = 6,721)	1[Reference]	1.27 (0.98–1.65)	1.09 (0.74–1.60)	1.14 (0.70–1.86)	2.28 (1.50–3.46)	0.001
Chronic lung disease (*N* = 9,187)	1[Reference]	1.19 (0.89–1.60)	1.22 (0.82–1.82)	0.84 (0.46–1.54)	0.88 (0.48–1.62)	0.939
Asthma (*N* = 10,040)	1[Reference]	1.14 (0.74–1.77)	1.42 (0.83–2.44)	0.57 (0.21–1.56)	1.49 (0.75–2.96)	0.421
Liver disease (*N* = 9,995)	1[Reference]	0.98 (0.67–1.46)	0.95 (0.54–1.66)	1.33 (0.71–2.51)	1.33 (0.70–2.51)	0.365
Cancer (*N* = 10,506)	1[Reference]	1.51 (0.88–2.57)	1.30 (0.59–2.88)	2.19 (0.97–4.92)	1.81 (0.70–4.66)	0.032
Digestive disease (*N* = 7,319)	1[Reference]	1.09 (0.82–1.46)	0.82 (0.51–1.31)	0.76 (0.40–1.43)	1.29 (0.75–2.23)	0.997
Kidney disease (*N* = 9,623)	1[Reference]	0.86 (0.58–1.26)	1.65 (1.10–2.48)	1.03 (0.53–1.99)	0.86 (0.43–1.73)	0.575
Arthritis (*N* = 5,791)	1[Reference]	1.16 (0.84–1.59)	1.16 (0.73–1.85)	1.00 (0.51–1.96)	0.88 (0.43–1.79)	0.796
Psychiatric disease (*N* = 10, 468)	1[Reference]	1.60 (0.95–2.68)	1.53 (0.77–3.02)	2.34 (1.13–4.82)	1.65 (0.69–3.95)	0.015
Memory-related disease (*N* = 10,424)	1[Reference]	1.34 (0.91–1.98)	1.45 (0.88–2.39)	2.60 (1.55–4.37)	1.94 (1.09–3.48)	< 0.001

^a^Models in overall population were fully adjusted for age, gender, marital status, educational level, rural or urban residence, smoking status, household wealth level, depressive symptoms, social isolation, and body mass index (BMI) category

^b^Reference: No Activity of Daily Living (ADL) Dependency

^c^Reference: No Instrumental Activity of Daily Living (IADL) Dependency

**Table 4:** Association between functional dependency level and incident chronic diseases in SHARE

	Adjusted odds ratio ^a^ (95%CI) by Functional Dependency Level					
Chronic disease	0	1	2	3	≥ 4	P-value for trend
ADL^b^ Dependency						
Hypertension (*N* = 19,149)	1[Reference]	1.33 (1.13–1.55)	1.28 (0.97–1.69)	1.59 (1.10–2.31)	1.35 (0.94–1.94)	< 0.001
Hypercholesterolemia (*N* = 24,128)	1[Reference]	1.16 (1.00–1.35.00.35)	1.25 (0.97–1.61)	1.79 (1.28–2.50)	1.34 (0.96–1.87)	< 0.001
Diabetes (*N* = 28,121)	1[Reference]	1.42 (1.17–1.71)	1.48 (1.07–2.05)	1.75 (1.13–2.70)	2.30 (1.58–3.35)	< 0.001
Heart disease (*N* = 27,950)	1[Reference]	1.71 (1.45–2.02)	2.06 (1.56–2.70)	3.21 (2.29–4.50)	2.81 (2.02–3.90)	< 0.001
Stroke (*N* = 30,721)	1[Reference]	1.71 (1.35–2.15)	1.92 (1.30–2.83)	3.61 (2.35–5.53)	3.46 (2.24–5.34)	< 0.001
Chronic lung disease (*N* = 29,968)	1[Reference]	1.65 (1.32–2.05)	1.75 (1.21–2.53)	1.79 (1.07–2.98)	2.87 (1.92–4.30)	< 0.001
Hip fracture (*N* = 31,226)	1[Reference]	2.71 (2.05–3.59)	3.99 (2.69–5.91)	2.61 (1.33–5.11)	4.84 (2.97–7.88)	< 0.001
Other fracture (*N* = 29,366)	1[Reference]	2.00 (1.62–2.48)	1.98 (1.38–2.85)	3.97 (2.67–5.88)	2.09 (1.31–3.35)	< 0.001
Arthritis (*N* = 23,884)	1[Reference]	1.73 (1.40–2.14)	2.85 (2.06–3.95)	2.54 (1.59–4.05)	2.05 (1.29–3.25)	< 0.001
Cataracts (*N* = 29,315)	1[Reference]	1.94 (1.67–2.27)	1.69 (1.28–2.23)	1.65 (1.12–2.44)	1.63 (1.11–2.39)	< 0.001
Parkinson disease (*N* = 31,693)	1[Reference]	2.61 (1.77–3.84)	4.86 (2.98–7.94)	3.18 (1.40–7.23)	6.43 (3.63–11.41)	< 0.001
Osteoporosis (*N* = 3,948)	1[Reference]	3.68 (2.79–4.85)	3.90 (2.29–6.62)	6.26 (3.11–12.61)	2.12 (1.02–4.43)	< 0.001
Alzheimer’s disease (*N* = 31,631)	1[Reference]	2.38 (1.90–2.97)	2.79 (1.97–3.96)	4.29 (2.85–6.47)	3.46 (2.22–5.37)	0.125
Cancer (*N* = 30,393)	1[Reference]	1.20 (0.94–1.53)	1.32 (0.89–1.97)	1.36 (0.78–2.39)	0.98 (0.53–1.79)	< 0.001
Digestive disease (*N* = 30,056)	1[Reference]	1.97 (1.53–2.53)	2.81 (1.94–4.08)	4.02 (2.58–6.27)	2.22 (1.29–3.82)	< 0.001
IADL^c^ Dependency						
Hypertension (*N* = 7,288)	1[Reference]	1.02 (0.79–1.31)	0.88 (0.56–1.38)	1.14 (0.62–2.09)	0.84 (0.41–1.73)	0.78
Hypercholesterolemia (*N* = 9,535)	1[Reference]	1.13 (0.89–1.43)	1.25 (0.86–1.81)	2.06 (1.29–3.29)	0.67 (0.32–1.42)	0.069
Diabetes (*N* = 11,120)	1[Reference]	1.00 (0.74–1.36)	0.96 (0.56–1.63)	1.38 (0.74–2.59)	1.16 (0.55–2.47)	0.499
Heart disease (*N* = 10,812)	1[Reference]	1.03 (0.78–1.37)	1.66 (1.09–2.54)	2.38 (1.43–3.98)	1.80 (0.98–3.33)	< 0.001
Stroke (*N* = 12,128)	1[Reference]	1.37 (0.96–1.96)	2.03 (1.21–3.38)	2.02 (0.99–4.13)	2.93 (1.47–5.81)	< 0.001
Chronic lung disease (*N* = 11,824)	1[Reference]	1.34 (0.94–1.89)	1.25 (0.69–2.28)	1.17 (0.51–2.70)	2.01 (0.96–4.22)	0.032
Hip fracture (*N* = 12,326)	1[Reference]	1.74 (1.12–2.71)	3.38 (1.94–5.87)	1.51 (0.54–4.19)	2.95 (1.25–6.99)	< 0.001
Other fracture (*N* = 11,210)	1[Reference]	1.85 (1.35–2.54)	1.67 (0.97–2.88)	3.42 (1.90–6.15)	2.24 (1.07–4.72)	< 0.001
Arthritis (*N* = 9,457)	1[Reference]	1.26 (0.91–1.75)	1.80 (1.09–2.97)	1.99 (0.98–4.05)	1.87 (0.81–4.29)	0.001
Cataracts (*N* = 11,440)	1[Reference]	1.51 (1.17–1.94)	1.66 (1.11–2.49)	1.12 (0.60–2.09)	0.99 (0.46–2.12)	0.021
Parkinson disease (*N* = 12,452)	1[Reference]	1.78 (0.90–3.53)	3.57 (1.58–8.07)	0.99 (0.13–7.24)	6.10 (2.35–15.89)	< 0.001
Osteoporosis (*N* = 2,067)	1[Reference]	2.96 (1.99–4.39)	2.08 (0.95–4.57)	5.39 (2.02–14.42)	3.09 (1.07–8.89)	< 0.001
Alzheimer’s disease (*N* = 12,549)	1[Reference]	1.37 (0.93–2.00.93.00)	1.56 (0.87–2.80)	2.02 (1.01–4.04)	1.70 (0.76–3.83)	0.005
Cancer (*N* = 11,883)	1[Reference]	1.04 (0.70–1.54)	1.35 (0.74–2.46)	1.85 (0.89–3.85)	0.76 (0.24–2.43)	0.313
Digestive disease (*N* = 11,685)	1[Reference]	1.58 (1.07–2.34)	2.47 (1.44–4.21)	3.14 (1.65–5.97)	1.74 (0.69–4.36)	< 0.001

^a^Models were adjusted for age, gender, marital status, educational level, rural or urban residence, smoking status, household wealth level, depressive symptoms, social isolation,, and body mass index (BMI) category

^b^Reference: No Activity of Daily Living (ADL) Dependency

^c^Reference: No Instrumental Activity of Daily Living (IADL) Dependency

## Discussion

In this study, our results, drawn from two nationally representative cohorts, suggest that cumulative FD level is associated with 7-year subsequent onset of multimorbidity and individual chronic diseases among middle-aged and older adults in China and European countries. Significant dose-response associations were identified between FD levels and incident heart disease, arthritis, stroke, psychiatric disease (Parkinson disease in SHARE) and memory-related disease (Alzheimer’s disease in SHARE) in both cohorts. Stronger associations with incident multimorbidity were observed in individuals aged 60 and above, living in rural areas, having depressive symptoms, having lower household wealth and experiencing social isolation among Chinese participants, whereas these associations were not observed among European participants, possibly due to the difference in health system structures or social and economic disparities.

To our knowledge, this is the first study to longitudinally examine the association between FD level and future incident multimorbidity among older adults. Previous research on the predictive value of physical function for developing multimorbidity was scarce, and prior studies were not longitudinal, potentially introducing bias related to reverse causality. Although two previous studies reported bidirectional associations between disability after at most four years [40, 41], they did not account for the severity of FD or consider specific forms of dependency in their analysis. Moreover, they did not progress further to the stratified assessments or the prospective associations between baseline FD and occurrence of individual chronic diseases.

Our findings align with previous research on the independent effect of FD level on future chronic diseases such as mental illness (memory-related disease, Parkinson’s disease, etc.) and cardiovascular diseases (heart disease, arthritis and stroke) [42, 43]. The consistent dose-response associations between FD level and outcomes in both China and HIC settings implies two potential mechanisms: Firstly, challenges in managing instrumental and social activities among the older adults might curtail their ability to navigate acute cardiovascular events effectively [42], potentially leading to discrepancies in care access and variations in hospitalization procedures. Secondly, dependence may be associated with psychosocial challenges, such as reduced self-efficacy or feelings of social disconnection, thereby compromising mental well-being and increasing susceptibility to psychiatric diseases.

Absence of consensus on the measurement of FD has been a major challenge in generating the estimates necessary to facilitate cross-national comparison. Additionally, the highly culturally sensitive nature of FD level assessment makes interpretation difficult [44]. Therefore, we used the number of difficulties on Katz and Lawton’s indices of ADLs and IADLs [44] reported by participants to measure FD level, as these constitute performance-based measures applicable in large populations from different regions [45]. 

Utilizing cross-cultural population-based cohorts, we investigated whether the effect of FD on incident multimorbidity differed in HICs and MIC and identified groups more susceptible to incident multimorbidity and chronic diseases in relation to FD levels. Aligned with previous research [46, 47], we found higher multimorbidity incidence rates in China compared to HICs. Within each cohort, rural participants and those with lower economic status exhibited increased multimorbidity incidence. In addition, among Chinese participants, having ≥ 4 FDs was significantly associated with incident multimorbidity (1.52, 95% CI [1. 37–1.69] for ADL, 1.96 [1.21–3.17] for IADL), while this effect was not observed among HICs participants. Conversely, having two FDs was linked to nearly double the risk of multimorbidity among HIC participants, but not among MIC participants.

The observed discrepancy in the association between FD and multimorbidity in European HICs and China may be attributed to differences in healthcare system infrastructure. China’s hospital-centric system often leads to higher diagnosis rates due to frequent appointments and numerous medication prescriptions, whereas European HICs prioritize patient-centered care to mitigate the segmentation of care and burden of illness caused by current disease-specific guidelines [48]. However, in Europe, reliance on a single healthcare professional may result in unmet needs among mildly dependent patients (i.e., individuals with one or two dependencies), elevating their risk of developing multimorbidity. In HICs, individuals with two dependencies may receive more comprehensive care, potentially leading to increased chronic disease diagnoses, while older adults in China with similar dependency levels might face barriers such as financial constraints or lack of awareness to pursue medical attention for their chronic ailments, resulting in underestimation of multimorbidity. Moreover, our findings suggest that individuals with greater wealth tend to have better healthcare access, enhancing diagnostic accuracy. In the future, a shift towards multimorbidity-focused healthcare models, like the cumulative complexity model [49] and burden of treatment theory [50], is essential globally. Supporting generalist clinicians in delivering personalized, continuous care, especially in socioeconomically deprived areas [51], is crucial. Further research on health system solutions to prevent and manage multimorbidity is urgently required.

Several mechanisms may explain the observed associations between FD and incident multimorbidity or chronic disease in later life. First, loss of independence in self-care activities could limit older adults’ social interactions, increasing susceptibility to behavioral health risks associated with multimorbidity. Additionally, dependent individuals may struggle to seek medical help and adhere to treatment regimens [52], exacerbating physical illness. Second, FD patients with depressive symptoms may experience higher psychological stress [53] and multimorbidity risks, potentially due to reduced physical activity and dysregulated cortisal levels [54, 55]. Moreover, depression has been associated with increased healthcare utilization [56, 57], including among multimorbid patients [58], which might result in a higher rate of multimorbidity diagnosis. Third, social isolation has been linked to lower quality-of-life and increased disease incidence [59, 60]. Our study found a significantly elevated risk of multimorbidity among individuals with 3 dependencies and social isolation (ADL: 4.04 [1.86–8.77]; IADL: 2.42 [1.00–5.85]), highlighting the importance of positive social interactions in mitigating psychological distress [61], thereby alleviating deleterious outcomes in FD patients. Moreover, FD patients might benefit from social contagion with healthy practices such as quitting smoking. Overall, addressing early-discovered FD in older adults is crucial, though further research is needed to elucidate the underlying mechanisms.

Strengths of our study include its longitudinal design, the use of two large nationally representative cohorts for cross-cultural comparison and reliability enhancement in both MIC and HIC settings, and exploration of various demographic and SES factors modifying the associations between FD and incident multimorbidity and chronic disease status. Additionally, we conceptualized FD both as a cumulative score based on the total number of ADL/IADL disabilities experienced to measure its severity, and as individual components to show associations of specific forms of FD with health outcomes. Our findings suggest that early identification of FD could allow clinicians and caregivers to implement targeted interventions, such as exercise programs, social support initiatives, and healthcare coordination, to prevent or mitigate the progression to multimorbidity among older adults, thereby improving their overall health-related quality of life and reducing disease-related disability burden [62–64]. 

Several limitations of this study should also be mentioned. First, self-reported chronic disease diagnosis may lead to underestimation of disease prevalence due to recall bias or limited health awareness among participants [65]. Additionally, self-reports may vary across countries and cultural contexts, potentially affecting cross-national comparisons. However, prior studies have demonstrated high agreement between self-reported prevalence and medical records for conditions like hypertension and cardiovascular diseases, but the concordance for other chronic conditions requires careful consideration [66–69]. Second, missing data on FD and chronic diseases could also introduce bias by reducing the representativeness of the analytic sample and potentially underestimating disease burden. To address this, we compared results obtained from complete-case analyses with those from multiple imputation, and the findings remained consistent (eTables 4 and 5), which supports the robustness of our conclusions. Third, while we controlled for numerous covariates, the influence of unknown or unmeasured time-varying factors cannot be entirely ruled out in this study. Fourth, as individuals with multimorbidity at baseline were excluded and only those who survived until follow-up were included, the possibility of selection and survival bias cannot be fully dismissed. Comparisons of included and excluded participants (eTables 6 and 7) showed that while some differences existed (e.g., age, marital status, and education), the groups were generally comparable. Moreover, any survival bias is likely to be conservative, as participants who died before follow-up would be expected to have higher FD and risk of incident multimorbidity [70]. Future cohort studies should address these limitations by improving study design and reducing nonresponse rates.

## Conclusions

The findings of this population-based longitudinal study provide compelling evidence of a dose-response association between FD level and increased risk of incident multimorbidity and chronic diseases among middle-aged and older individuals in China and Europe. These associations were particularly pronounced among older individuals, rural residents, those with depressive symptoms, lower household wealth, and social isolation among MIC participants. Conversely, such associations were not evident in HICs, potentially due to difference in health system structure and social- economic disparities. Tailored interventions for individuals with specific FD levels or items are crucial to mitigate associated health risks and to support healthy aging and FD. Additionally, healthcare reforms should prioritize resource allocation and introduce integrated care models to address disparities in healthcare delivery.

## Supplementary Information

Supplementary Material 1.

## Data Availability

The datasets generated and analyzed during the current study are available in the CHARLS and SHARE website, available in http://charls.pku.edu.cn/en and [https://share-eric.eu/data/](https:/share-eric.eu/data).

## Abbreviations

ADL: Activities of daily living
IADL: Instrumental activities of daily living
FD: Functional dependency
OR: Odds ratio
AOR: Adjusted odds ratio
BMI: Body mass index
CHARLS: China health and retirement longitudinal study
SHARE: Survey of health, ageing and retirement in europe
CI: Confidence interval
HICs: High–income countries
MICs: Middle–income countries
NCD: Non–communicable disease
MICE: Multiple imputation by chained equations
SD: Standard deviation
SES: Socioeconomic status
