# Supplementary material for: Functional dependency level and later-life incident multimorbidity in older people: longitudinal evidence from 19 countries in China and Europe
Source: BMC Public Health. 2025 Dec 24;25:4292. doi: 10.1186/s12889-025-25259-7 (PMC12729261; doi:10.1186/s12889-025-25259-7)
Supplement: Supplementary file 1 — Supplementary Material 1. [file 12889_2025_25259_MOESM1_ESM.docx]

**Supplement Materials**

**Functional Dependency Level and Later-life Incident Multimorbidity in older people: Longitudinal evidence from 19 countries in China and Europe**

**eMethods. Participant selection and measurements and definitions**

**eTable 1. Proportion of Missing Data Before Imputation**

**eTable 2. Characteristics of CHARLS participants by ADL/IADL dependency status**

**eTable 3. Characteristics of SHARE participants by ADL/IADL dependency status**

**eTable 4. Association between functional dependency level and incident multimorbidity in the overall cohort and subgroups in CHARLS, with imputed data sets**

**eTable 5 Association between functional dependency level and incident multimorbidity in the overall cohort and subgroups in SHARE, with imputed data sets**

**eTable 6. Comparison of baseline characteristics between included and excluded participants in CHARLS cohort.**

**eTable 7. Comparison of baseline characteristics between included and excluded participants in SHARE cohort.**

**eFigure 1. Study flowchart of participant selection**

**eFigure 2. Association between individual functional dependency indicator and incident multimorbidity**

**eFigure 3. Combined associations between level of functional dependency and follow-up incident multimorbidity**

**eReferences**

**eMethods. Participant selection and measurements and definitions**

**Participants Selection**

The detailed process of participant selection in each cohort is described in **eFigure 1**. The baseline sample in CHARLS comprised 17,708 participants from 450 villages or resident communities in 28 provinces across China. Respondents were followed up every two years, and a small pool of new participants was recruited in every survey wave. Among participants, 13,175 individuals remained in the 2018 follow-up wave and had their chronic disease status measured. After removing 2,663 individuals with missing information on any of the 11 FD components and 12 individuals without any data on chronic disease status, 10,500 participants with information on at least one of the 14 chronic diseases were remained. Furthermore, participants with all information on 14 chronic diseases at baseline and follow-up, and have multimorbidity at baseline were excluded. Finally 6,773 and 6,692 CHARLS participants were eligible for the main analyses of associaitons between ADL and IADL dependency with incident multimorbidity, respectively. In the subsequent analysis of FDs and incident chronic diseases, we re-included individuals who did not have the specific chronic disease at baseline, had no missing data for all covariates, and were followed up for 7 years.

SHARE is a large multicultural, cross-sectional, publicly available data set from a survey modeled after the Health and Retirement Study in the United States. SHARE includes data on health, socio-economic characteristics, family and social relationships, and mental health from individuals aged 50 years and older from 18 European countries (14 in Wave 2) as well as Israel. The Wave 4 (2011) sample in SHARE comprised 58,121 individuals. Among then, 32,113 individuals remained in the 2017 follow-up wave and had their chronic disease status measured. After removing individuals without any data on chronic disease status and individuals with missing information on any of the 16 FD components, participants with information on at least one of the 16 chronic diseases were included to evaluate longitudinal associations between FD levels and chronic diseases. Furthermore, participants with all information on 15 chronic diseases at baseline and follow-up, and have multimorbidity at baseline were excluded, resulting in 6,574 participants were eligible for the main analyses of ADL and IADL dependency, respectively. Similarly, in the subsequent analysis of FDs and incident chronic diseases, we re-included individuals who did not have the specific chronic disease at baseline, had no missing data for all covariates, and were followed up till 2017. This approach ensures a comprehensive examination of the associations between FD and the development of new chronic conditions over time.

**Measurements and Definitions**

Covariates were identified through literature reviews. Data on demographics (age and gender), socio-economic characteristics (education, living region, and household wealth level), living arrangements (marital status), lifestyles (smoking and BMI category), mental symptoms (depressive symptoms), and social isolation were collected. Household wealth was calculated by summing all wealth components including residence, vehicles, and savings accounts in local currencies and excluding other debts at the couple level. We used tertiles of total household wealth in each study to make the measure comparable across studies, with 0 to 2 representing the lowest (quartile 1) to the highest (quartile 3). The index of social isolation was generated based on social networks and social activity or engagement. The index was assigned one point if participants were not married (never married, separated, divorced, widowed), living alone, having less than weekly contact (including face-to-face, telephone, or e-mail) with their children, living in a rural area, or not participating in any social activities (sports or social clubs, community-related organizations, interacting with friends, providing help, voluntary or charity work, using internet, playing mah-jong or cards, attending an educational or training course, or stock investment)^2^. A total social isolation score ranging from zero to five was calculated by summing these points, with higher scores indicating greater social isolation. We therefore categorized participants according to whether their scores were low (< 2) or high (≥2)^3^.

Residential area was classified as living in an urban or suburb area (a big city, a suburb or the outskirts of a big city) , living in town (a large/small town) or living in rural area (a rural area or village) in SHARE. In CHARLS, the first two classes were group into one as “Urban”. Marital status was classified as married (married or partnered) or bachelordom (separated, divorced, widowed, or never married). Educational level was categorized into three classes: less than no formal education/illiterate, primary school or below, or middle school or above. Smoking status was classified as never, past, or current smoker. BMI calculated through body weight in kilogrammes divided by height squared (kg/m^2^) was categorized into underweight (BMI < 18.5), normal (18.5 ≤ BMI < 24), overweight (24 ≤ BMI < 28), and obese (BMI ≥ 28) according to the China Ministry of Health Disease Control Division ^5^ in CHARLS. In SHARE, BMI categories were calculated in accordance with World Health Organization guidelines:^6^ normal weight (18.5–24.9 kg/m^2^), overweight (25–29.9 kg/m^2^), and obese (≥30 kg/m^2^). Depressive symptoms were measured based on the Center for Epidemiological Studies Depression Scale (CES-D-10) using ten questions in CHARLS, and a cut-off score of ≥10 (range: 0-30) was used to distinguish participants with depression from those relatively free of depression.^7^ In SHARE, depression symptoms were assessed based on the EURO-D 12-item scale, and an optimal cut-off point of ≥4 was used to diagnose a clinically significant case of depression.^8^

**eTable 1. Proportion of Missing Data Before Imputation.**

| **Variable** | **CHARLS (N=17,708)** | **SHARE (N=58,121)** | **Category** |
| --- | --- | --- | --- |
| **Functional Dependency（FD, 2011)** |  |  |  |
| ADL | 0.7% | 0.1% | FD |
| IADL | 0.7% | 0.1% | FD |
| **Demographics (2011)** |  |  |  |
| Age | – | – | Demographics |
| Male | – | – | Demographics |
| **Socioeconomic (2011)** |  |  |  |
| Marital Status | – | 33.7% | Socio-demographic |
| Education Level | 0.1% | 2.2% | Socio-demographic |
| Urban Residence | – | 33.9% | Socio-demographic |
| Household Wealth | 0.9% | 0.2% | Socio-economic |
| **Lifestyle (2011)** |  |  |  |
| BMI Category | 19.9% | 5.1% | Lifestyle |
| Smoking Status | 3.9% | 36.2% | Lifestyle |
| **Mental & Social (2011)** |  |  |  |
| Depressive Symptoms | – | 1.9% | Mental Health |
| Social Isolation | – | 55.4% | Social Engagement |
| **Chronic Diseases at follow-ups** |  |  |  |
| Hypertension | 45.8% | 43.9% | Non-Communicable Diseases (NCD) |
| Dyslipidemia / High Cholesterol | 33.2% | 26.8% | NCD |
| Diabetes | 29.1% | 13.6% | NCD |
| Heart Disease | 34.1% | 12.5% | NCD |
| Stroke | 24.8% | 5.9% | NCD |
| Chronic Lung Disease | 30.9% | 7.1% | NCD |
| Asthma | 26.4% | – | NCD |
| Liver Disease | 25.9% | – | NCD |
| Cancer | 23.6% | 4.1% | NCD |
| Digestive Disease | 41.7% | 8.4% | NCD |
| Kidney Disease | 28.0% | – | NCD |
| Arthritis / Rheumatism | 50.4% | 23.1% | NCD |
| Psychiatric Disease | 24.0% | – | NCD |
| Memory-related Disease | 24.4% | – | NCD |
| Osteoporosis | – | 6.3% | NCD |
| Cataracts | – | 15.8% | NCD |
| Parkinson’s Disease | – | 1.0% | NCD |
| Alzheimer’s Disease | – | 0.9% | NCD |
| Hip Fracture | – | 2.0% | NCD |
| Other Fractures | – | 3.4% | NCD |

**eTable 2. Characteristics of CHARLS participants by ADL/IADL dependency status**

|  | ADL  Dependency,  (N = 6773) |  |  | IADL  Dependency,  (N = 6692) |  |  |  |
| --- | --- | --- | --- | --- | --- | --- | --- |
| Characteristics | No  (n = 2975) | At least  one (n=3798) | P Value | No  (n=5750) | At least  one  (n=942) | P Value |  |
| Age | 55.71  (7.80) | 59.18  (9.33) | <.001 | 57.02  (8.52) | 61.15  (9.72) | <.001 |  |
| Gender |  |  | <.001 |  |  | <.001 |  |
| Male | 1,744 (58.62) | 1,516 (39.92) |  | 2,872 (49.95) | 358  (38.00) |  |  |
| Female | 1,231 (41.38) | 2,282 (60.08) |  | 2,878 (50.05) | 584  (62.00) |  |  |
| Marital status |  |  | <.001 |  |  | <.001 |  |
| Married | 2,631 (88.44) | 3,141 (82.70) |  | 4,953 (86.14) | 755  (80.15) |  |  |
| Unmarried | 344  (11.56) | 657  (17.30) |  | 797  (13.86) | 187  (19.85) |  |  |
| Education, N (%) |  |  | <.001 |  |  | <.001 |  |
| No formal education/illiterate | 996  (33.48) | 1,990 (52.40) |  | 2,327 (40.47) | 618  (65.61) |  |  |
| Primary school or below | 682  (22.92) | 829  (21.83) |  | 1,313 (22.83) | 187  (19.85) |  |  |
| Middle school or above | 1,297 (43.60) | 979  (25.78) |  | 2,110 (36.70) | 137  (14.54) |  |  |
| Area of residence, N (%) |  |  | <.001 |  |  | <.001 |  |
| Rural | 1,820 (61.18) | 2,617 (68.90) |  | 3,670 (63.83) | 710  (75.37) |  |  |
| Urban | 1,155 (38.82) | 1,181 (31.10) |  | 2,080 (36.17) | 232  (24.63) |  |  |
| Smoking status, N (%) |  |  | 0.001 |  |  | 0.001 |  |
| Never smoker | 1,643 (55.23) | 2,408 (65.30) |  | 3,439 (59.81) | 623  (66.14) |  |  |
| Former smoker | 201  (6.76) | 242  (6.37) |  | 380  (6.61) | 61  (6.48) |  |  |
| Current smoker | 1,131 (38.02) | 1,076 (28.33) |  | 1,931 (33.58) | 258  (27.39)) |  |  |
| BMI, N (%) |  |  | <.001 |  |  | <.001 |  |
| Underweight (<18.5) | 120  (4.03) | 284  (7.48) |  | 310  (5.39) | 91  (9.66) |  |  |
| Normal (18.5–24.0) | 1,736 (58.35) | 2,023 (53.26) |  | 3,186 (55.41) | 534  (56.69) |  |  |
| Overweight (24.0–28.0) | 862  (28.97) | 1,023 (26.94) |  | 1,644 (28.59) | 218  (23.14) |  |  |
| Obese (≥28.0) | 257  (8.64) | 468  (12.32) |  | 610  (10.61) | 99  (10.51) |  |  |
| Household Wealth Level |  |  | <.001 |  |  | 0.132 |  |
| Low | 865  (29.08) | 1,275 (33.57) |  | 1,778 (30.92) | 320  (33.97) |  |  |
| Medium | 947  (31.83) | 1,205 (31.73) |  | 1,832 (31.86) | 297  (31.53) |  |  |
| High | 1,163 (39.09) | 1,318 (34.70) |  | 2,140 (37.22) | 325  (34.50) |  |  |
| Depressive Symptoms |  |  | <.001 |  |  | <.001 |  |
| Yes | 590  (19.83) | 1,631 (42.94) |  | 1,663 (28.92) | 526  (55.84) |  |  |
| No | 2,385 (80.17) | 2,167 (57.06) |  | 4,087 (71.08) | 416  (44.16) |  |  |
| Social Isolation |  |  | <.001 |  |  | <.001 |  |
| Non-isolated | 1,421 (47.76) | 1,360 (35.81) |  | 2,469 (42.94) | 283  (30.04) |  |  |
| Isolated | 1,554 (52.24) | 2,438 (64.19) |  | 3,281 (57.06) | 659  (69.96) |  |  |
| Chronic disease, N (%) (Following up) |  |  |  |  |  |  |  |
| Hypertension | 464  (15.47) | 190  (17.86) | 0.016 | 701  (15.17) | 236  (16.32) | 0.055 |  |
| Dyslipidemia | 366  (10.49) | 168  (11.31) | <.001 | 482  (9.07) | 209  (10.71) | 0.952 |  |
| Diabetes | 199  (5.51) | 118  (7.21) | <.001 | 268  (4.89) | 142  (6.57) | 0.048 |  |
| Heart disease | 265  (7.68) | 156  (11.30) | <.001 | 336  (6.26) | 190  (10.25) | <.001 |  |
| Stroke | 167  (4.46) | 152  (8.71) | <.001 | 239  (4.20) | 189  (8.19) | <.001 |  |
| Chronic lung disease | 176  (5.00) | 121  (8.01) | <.001 | 240  (4.43) | 144  (7.12) | <.001 |  |
| Asthma | 63  (1.70) | 55  (3.19) | <.001 | 79  (1.40) | 75  (3.30) | <.001 |  |
| Liver disease | 90  (2.43) | 64  (3.70) | 0.045 | 142  (2.53) | 76  (3.35) | 0.513 |  |
| Cancer | 56  (1.49) | 30  (1.61) | 0.079 | 60  (1.05) | 45  (1.84) | 0.011 |  |
| Digestive disease | 258  (8.17) | 102  (9.49) | 0.001 | 346  (7.09) | 132  (8.99) | 0.130 |  |
| Kidney disease | 117  (3.20) | 91  (5.71) | <.001 | 161  (2.90) | 99  (4.58) | 0.063 |  |
| Arthritis | 292  (10.74) | 67  (11.57) | <.001 | 404  (9.32) | 110  (10.95) | 0.028 |  |
| Psychiatric disease | 40  (1.06) | 49  (2.69) | <.001 | 41  (0.72) | 58  (2.45) | <.001 |  |
| Memory-related disease | 85  (2.27) | 87  (4.91) | <.001 | 84  (1.48) | 113  (4.83) | <.001 |  |
| Multimorbidity, N (%)  (Following up) | 1,882 (49.53) | 465  (60.31) | <.001 | 2,478 (43.10) | 597  (52.46) | <.001 |  |

**eTable 3. Characteristics of SHARE participants by ADL/IADL dependency status**

|  | ADL  Dependency,  (N =6574) |  |  | IADL  Dependency,  (N = 6574) |  |  |
| --- | --- | --- | --- | --- | --- | --- |
| Characteristics | No  (n = 6334) | At least one  (n=240) | P  Value | No  (n =6119) | At least one (n=455) | P Value |
| Age | 61.77  (8.54) | 64.18 (10.47) | <.001 | 61.54  (8.35) | 66.17 (10.87) | <.001 |
| Gender |  |  | 0.492 |  |  | <.001 |
| Male | 2,675  (42.23) | 96  (40.00) |  | 2,649 (43.29) | 122 (26.81) |  |
| Female | 3,659  (57.77) | 144 (60.00) |  | 3,470 (56.71) | 333 (73.19) |  |
| Marital status |  |  | 0.002 |  |  | <.001 |
| Married | 2,327  (36.74) | 112 (46.67) |  | 2,214 (36.18) | 225 (49.45) |  |
| Unmarried | 4,007  (63.26) | 128 (53.33) |  | 3,905 (63.82) | 230 (50.55) |  |
| Education, N (%) |  |  | <.001 |  |  | <.001 |
| No formaleducation/illiterate | 1,808  (28.54) | 105 (43.75) |  | 1,687 (27.57) | 226 (49.67) |  |
| Primary school or below | 2,874  (45.37) | 104 (43.33) |  | 2,810 (45.92) | 168 (36.92) |  |
| Middle school or above | 1,652  (26.08) | 31  (12.92) |  | 1,622 (26.51) | 61  (13.41) |  |
| Area of residence, N (%) |  |  | 0.095 |  |  | 0.012 |
| Urban or Suburbs | 1,435  (23.92) | 46  (19.83) |  | 1,394 (24.09) | 87  (19.59) |  |
| Town | 2,159  (36.00) | 77  (33.19) |  | 2,086 (36.05) | 150 (33.78) |  |
| Rural area | 2,404  (40.08) | 109 (46.98) |  | 2,306 (39.85) | 207 (46.62) |  |
| Smoking status, N (%) |  |  | 0.847 |  |  | 0.043 |
| Never smoker | 3,566  (56.30) | 134 (55.83) |  | 3,428 (56.02) | 272 (59.78) |  |
| Former smoker | 1,460  (23.05) | 53  (22.08) |  | 1,430 (23.37) | 83  (18.24) |  |
| Current smoker | 1,308  (20.65) | 53  (22.08) |  | 1,261 (20.61) | 100 (21.98) |  |
| BMI, N (%) |  |  | <.001 |  |  | 0.134 |
| Underweight (<18.5) | 62  (0.98) | 1  (0.42) |  | 59  (0.96) | 4  (0.88) |  |
| Normal (18.5–24.0) | 2,635  (41.60) | 70  (29.17) |  | 2,528 (41.31) | 177 (38.90) |  |
| Overweight(24.0–28.0) | 2,613  (41.25) | 96  (40.00) |  | 2,529 (41.33) | 180 (39.56) |  |
| Obese (≥28.0) | 1,024  (16.17) | 73  (30.42) |  | 1,003 (16.39) | 94 (20.66) |  |
| Household Wealth, N (%) |  |  | <.001 |  |  | <.001 |
| Low | 1,994  (31.57) | 106 (44.17) |  | 1,873 (30.69) | 227 (49.89) |  |
| Medium | 2,248  (35.59) | 84  (35.00) |  | 2,189 (35.87) | 143 (31.43) |  |
| High | 2,075  (32.85) | 50  (20.83) |  | 2,040 (33.43) | 85  (18.68) |  |
| Social Isolation |  |  | <.001 |  |  | <.001 |
| Non-isolated | 3,288  (54.82) | 94  (40.52) |  | 3,205 (55.39) | 177 (39.86) |  |
| Isolated | 2,710  (45.18) | 138 (59.48) |  | 2,581 (44.61) | 267 (60.14) |  |
| Chronic disease, N (%) (Following up) |  |  |  |  |  |  |
| Hypertension | 4,261  (23.88) | 388 (29.66) | <.001 | 4,041 (23.66) | 608 (29.41) | <.001 |
| Hypercholesterolemia | 3,567  (16.09) | 380 (19.36) | <.001 | 3,319 (15.77) | 628 (20.40) | <.001 |
| Diabetes | 1,646  (6.38) | 223 (9.53) | <.001 | 1,535  (6.26) | 334  (9.27) | <.001 |
| Heart disease | 2,041  (7.93) | 328 (14.75) | <.001 | 1,912  (7.78) | 457 (13.48) | <.001 |
| Stroke | 849  (3.02) | 156  (6.05) | <.001 | 756  (2.83) | 249  (6.22) | <.001 |
| Chronic lung disease | 1,004  (3.67) | 166  (6.43) | <.001 | 922  (3.55) | 248  (6.22) | <.001 |
| Hip fracture | 383  (1.34) | 114  (4.15) | <.001 | 354  (1.31) | 143  (3.38) | <.001 |
| Other fracture | 920  (3.43) | 182  (7.19) | <.001 | 856  (3.37) | 246  (6.24) | <.001 |
| Arthritis | 1,518  (6.77) | 189 (12.84) | <.001 | 1381  (6.44) | 326 (13.38) | <.001 |
| Cataracts | 2,011  (7.50) | 323 (12.93) | <.001 | 1,881  (7.37) | 453 (11.99) | <.001 |
| Parkinson’s disease | 194  (0.67) | 67  (2.32) | <.001 | 180  (0.66) | 81  (1.83) | <.001 |
| Osteoporosis | 550  (15.33) | 145 (40.17) | <.001 | 504  (14.99) | 191 (32.65) | <.001 |
| Alzheimer's disease | 670  (2.33) | 176  (6.15) | <.001 | 559  (2.05) | 287  (6.61) | <.001 |
| Digestive disease | 629  (2.29) | 137  (5.22) | <.001 | 581  (2.23) | 185  (4.57) | <.001 |
| Cancer | 1,019  (3.69) | 122  (4.44) | 0.047 | 960  (3.66) | 181  (4.32) | 0.036 |
| Multimorbidity, N (%)  (Following up) | 5,833  (35.92) | 360 (56.78) | <.001 | 5,596 (35.52) | 597 (53.40) | <.001 |

**eTable 4. Association between functional dependency level and incident multimorbidity in the overall cohort and subgroups in CHARLS, with imputed data sets**

|  | ADL^a^ |  | |  | |  | |  | |  | | IADL^b^ |  | |  | | | |  | |  |  |
| --- | --- | --- | --- | --- | --- | --- | --- | --- | --- | --- | --- | --- | --- | --- | --- | --- | --- | --- | --- | --- | --- | --- |
|  | 1^c^ | 2 | | 3 | | ≥4 | | P  value | | P  value | | 1 | 2 | | 3 | | | ≥4 | | | P  value | P  value |
|  |  |  | |  | |  | | for trend | | for interaction  in ADL | | |  | |  | |  | | | | for  trend | for interaction in IADL |
| Overall | 1.26  (1.15-1.37) | 3.17  (2.62-3.84) | | 1.06  (0.83-1.37) | | 0.80  (0.65-0.98) | | <.001 | | <.001 | | 1.11  (1.07-1.16) | 1.14  (1.07-1.21) | | 1.53  (1.39-1.69) | | 2.60  (2.32-  2.91) | | | | <.001 | <.001 |
| Subgroup |  |  | |  | |  | |  | |  | |  |  | |  | |  | | | |  |  |
| Age |  |  | |  | |  | |  | |  | |  |  | |  | |  | | | |  |  |
| <60 | 1.53  (1.44-1.64) | 2.22  (1.96-2.53) | | 4.53  (3.58-5.72) | | 1.62  (1.39-1.89) | | <.001 | | <.001 | | 1.18  (1.12-1.25) | 1.58  (1.45-1.73) | | 1.69  (1.42-2.01) | | 2.40  (2.00-  2.90) | | | | <.001 | <.001 |
| ≥60 | 1.72  (1.61-1.83) | 1.23  (1.11-1.36) | | 2.58  (2.20-3.02) | | 2.08  (1.83-2.36) | | <.001 | | <.001 | | 1.08  (1.02-1.15) | 0.92  (0.85-1.00) | | 1.50  (1.34-1.69) | | 3.02  (2.61-  3.49) | | | | <.001 | <.001 |
| Gender |  |  | |  | |  | |  | |  | |  |  | |  | |  | | | |  |  |
| Male | 1.40  (1.30-1.50) | 1.86  (1.64-2.11) | | 2.77  (2.24-3.43) | | 2.00  (1.70-2.35) | | <.001 | | <.001 | | 0.92  (0.87-0.98) | 0.94  (0.85-1.04) | | 2.48  (2.09-2.94) | | 2.40  (2.03-  2.85) | | | | <.001 | <.001 |
| Female | 1.74  (1.64-1.84) | 1.27  (1.14-1.40) | | 2.87  (2.43-3.40) | | 1.57  (1.39-1.78) | | <.001 | | <.001 | | 1.27  (1.21-1.34) | 1.26  (1.16-1.35) | | 1.17  (1.04-1.32) | | 2.77  (2.37-  3.24) | | | | <.001 | <.001 |
| Residence Area | | |  | |  | |  | |  | |  |  | |  | |  | | | |  |  |  |
| Urban | 1.41  (1.29-1.53) | 1.27  (1.09-1.48) | | 2.92  (2.20-3.88) | | 0.96  (0.78-1.20) | | <.001 | | <.001 | | 1.03  (0.95-1.12) | 1.14  (1.01-1.28) | | 0.75  (0.62-  0.92) | | | | 1.35  (1.10-1.65) | | 0.076 | <.001 |
| Rural | 1.71  (1.62-1.80) | 1.58  (1.44-1.73) | | 2.84  (2.45-3.30) | | 2.06  (1.84-2.31) | | <.001 | | <.001 | | 1.15  (1.10-1.20) | 1.14  (1.06-1.22) | | 1.88  (1.68-  2.10) | | | | 3.49  (3.03-4.02) | | <.001 | <.001 |
| Marital status |  |  | |  | |  | |  | |  | |  |  | |  | | | |  | |  |  |
| Married | 1.52  (1.45-1.60) | 1.65  (1.51-1.80) | | 3.03  (2.61-3.52) | | 1.73  (1.55-1.94) | | <.001 | | <.001 | | 1.10  (1.05-1.15) | 1.33  (1.24-1.42) | | 1.54  (1.38-  1.71) | | | | 2.67  (2.34-3.06) | | <.001 | <.001 |
| Bachelordom | 2.07  (1.83-2.34) | 0.93  (0.75-1.16) | | 2.79  (2.09-3.71) | | 2.01  (1.62-2.48) | | <.001 | | <.001 | | 1.14  (1.02-1.28) | 0.60  (0.52-0.70) | | 1.57  (1.26-  1.94) | | | | 2.55  (2.04-3.19) | | <.001 | <.001 |
| Depressive Symptoms | | |  | |  | |  | |  | |  |  | |  | |  | | | |  |  |  |
| No | 1.56  (1.45-1.68) | 1.83  (1.55-2.15) | | 7.78  (5.60-10.80) | | 1.11  (0.89-1.38) | | <.001 | | <.001 | | 0.92  (0.86-0.98) | 1.05  (0.93-1.17) | | 1.71  (1.41-  2.07) | | | | 4.03  (3.16-5.15) | | <.001 | <.001 |
| Yes | 1.62  (1.52-1.71) | 1.39  (1.26-1.52) | | 2.19  (1.89-2.54) | | 2.03  (1.81-2.27) | | <.001 | | <.001 | | 1.26  (1.19-1.33) | 1.19  (1.10-1.27) | | 1.50  (1.34-  1.68) | | | | 2.34  (2.05-2.66) | | <.001 | <.001 |
| Household Wealth Level | | |  | |  | |  | |  | |  |  | |  | |  | | | |  |  |  |
| Low | 1.77  (1.64-1.91) | 1.08  (0.96-1.21) | | 4.18  (3.35-5.22) | | 2.76  (2.32-3.28) | | <.001 | | <.001 | | 1.15  (1.07-1.23) | 1.02  (0.91-1.13) | | 1.44  (1.25-  1.66) | | | | 5.48  (4.42-6.81) | | <.001 | <.001 |
| Medium | 1.74  (1.61-1.88) | 1.56  (1.35-1.80) | | 4.32  (3.39-5.51) | | 1.83  (1.57-2.14) | | <.001 | | <.001 | | 0.99  (0.93-1.07) | 1.45  (1.30-1.62) | | 1.52  (1.27-  1.81) | | | | 1.87  (1.54-2.26) | | <.001 | <.001 |
| High | 1.26  (1.15-1.37) | 3.17  (2.62-3.84) | | 1.06  (0.83-1.37) | | 0.80  (0.65-0.98) | | <.001 | | <.001 | | 1.17  (1.09-1.25) | 1.06  (0.96-1.18) | | 1.74  (1.43-  2.12) | | | | 1.53  (1.24-1.88) | | <.001 | <.001 |
| Social Isolation | | |  | |  | |  | |  | |  |  | |  | |  | | | |  |  |  |
| No | 1.54  (1.43-1.65) | 1.27  (1.12-1.45) | | 1.27  (1.06-1.53) | | 3.20  (2.62-3.90) | | <.001 | | <.001 | | 1.18  (1.11-1.25) | 0.98  (0.88-1.08) | | 1.50  (1.28-  1.75) | | | | 2.56  (2.02-3.26) | | <.001 | <.001 |
| Yes | 1.65  (1.56-1.75) | 1.68  (1.51-1.86) | | 6.48  (5.20-8.08) | | 1.42  (1.27-1.59) | | <.001 | | <.001 | | 1.05  (0.99-1.11) | 1.27  (1.17-1.37) | | 1.62  (1.43-  1.83) | | | | 2.61  (2.29-2.97) | | <.001 | <.001 |

1. ADL: Activity of Daily Living.
2. IADL: Instrumental Activity of Daily Living.
3. Reference: No Functional Dependency.
4. Odds Ratio (95% CI). Models in overall population were fully adjusted for age, gender, marital status, educational level, rural or urban residence, smoking status, household wealth level, depressive symptoms, social isolation, and body mass index (BMI) category. In subgroup analysis, all other covariates were adjusted for except the stratified variable.

**eTable 5. Association between functional dependency level and incident multimorbidity in the overall cohort and subgroups in SHARE, with imputed data sets**

|  | ADL^a^ |  |  |  |  |  | IADL^b^ |  |  |  |  |  | |
| --- | --- | --- | --- | --- | --- | --- | --- | --- | --- | --- | --- | --- | --- |
|  | 1^c^ | 2 | 3 | ≥4 | P value | P value | 1 | 2 | 3 | ≥4 | P value | P value |  |
|  |  |  |  |  | for trend | for interaction  in ADL | |  |  |  | for trend | for interaction  in IADL | |
| Overall | 1.94  (1.85-2.03) | 2.08  (1.88-2.30) | 1.22  (1.01-1.47) | 1.33  (1.14-1.56) | <.001 | <.001 | 1.56  (1.51-1.61) | 2.28  (2.13-2.45) | 1.66  (1.43-1.93) | 1.35  (1.21-1.51) | <.001 | <.001 | |
| Subgroup |  |  |  |  |  |  |  |  |  |  |  |  | |
| Age |  |  |  |  |  |  |  |  |  |  |  |  | |
| <60 | 1.61  (1.50-1.74) | 2.92  (2.52-3.39) | 2.54  (1.86-3.47) | 1.99  (1.55-2.56) | <.001 | <.001 | 1.80  (1.70-1.90) | 2.85  (2.53-3.21) | 4.11  (2.76-6.13) | 5.80  (4.48-7.51) | <.001 | <.001 | |
| ≥60 | 2.43  (2.29-2.58) | 1.83  (1.61-2.07) | 0.96  (0.76-1.21) | 1.10  (0.90-1.33) | <.001 | <.001 | 1.69  (1.62-1.76) | 2.44  (2.24-2.65) | 2.02  (1.73-2.36) | 1.20  (1.07-1.35) | <.001 | <.001 | |
| Gender |  |  |  |  |  |  |  |  |  |  |  |  | |
| Male | 1.63  (1.51-1.75) | 2.28  (1.94-2.68) | 1.06  (0.78-1.43) | 1.67  (1.39-2.01) | <.001 | <.001 | 1.68  (1.57-1.80) | 2.68  (2.34-3.07) | 2.71  (2.10-3.48) | 1.67  (1.39-2.01) | <.001 | <.001 | |
| Female | 2.14  (2.01-2.27) | 1.96  (1.73-2.23) | 1.37  (1.06-1.77) | 0.86  (0.65-1.14) | <.001 | <.001 | 1.49  (1.43-1.55) | 2.16  (2.00-2.35) | 1.28  (1.06-1.54) | 1.18  (1.03-1.35) | <.001 | <.001 | |
| Residence Area | |  |  |  |  |  |  |  |  |  |  |  | |
| Urban | 2.46  (2.21-2.74) | 4.80  (3.88-5.95) | 2.42  (1.62-3.62) | 0.91  (0.68-1.21) | <.001 | <.001 | 1.48  (1.37-1.60) | 2.07  (1.78-2.41) | 1.91  (1.29-2.82) | 2.04  (1.58-2.64) | <.001 | <.001 | |
| Rural | 2.16  (2.02-2.31) | 1.37  (1.17-1.60) | 0.67  (0.47-0.94) | 3.27  (2.29-4.67) | <.001 | <.001 | 1.50  (1.42-1.58) | 3.33  (2.98-3.71) | 1.77  (1.47-2.13) | 1.67  (1.39-2.00) | <.001 | <.001 | |
| Town | 1.49  (1.37-1.61) | 1.93  (1.62-2.30) | 1.34  (1.01-1.79) | 1.24  (0.99-1.55) | <.001 | <.001 | 1.72  (1.62-1.82) | 1.61  (1.44-1.81) | 0.91  (0.64-1.27) | 1.03  (0.87-1.22) | <.001 | <.001 | |
| Marital status |  |  |  |  |  |  |  |  |  |  |  |  | |
| Married | 2.27  (2.13-2.41) | 2.43  (2.09-2.81) | 0.85  (0.66-1.08) | 0.96  (0.79-1.17) | <.001 | <.001 | 1.68  (1.60-1.76) | 3.45  (3.08-3.86) | 6.38  (4.86-8.38) | 1.05  (0.88-1.25) | <.001 | <.001 | |
| Bachelordom | 1.57  (1.47-1.69) | 1.77  (1.55-2.02) | 2.21  (1.58-3.09) | 2.38  (1.82-3.10) | <.001 | <.001 | 1.41  (1.34-1.49) | 1.67  (1.53-1.83) | 0.55  (0.45-0.68) | 1.62  (1.41-1.87) | <.001 | <.001 | |
| Depressive Symptoms | |  |  |  |  |  |  |  |  |  |  |  | |
| No | 1.80  (1.68-1.93) | 2.49  (2.10-2.95) | 0.43  (0.26-0.70) | 0.88  (0.71-1.08) | <.001 | <.001 | 1.64  (1.56-1.72) | 2.78  (2.48-3.12) | 2.04  (1.64-2.54) | 1.24  (1.07-1.44) | <.001 | <.001 | |
| Yes | 2.04  (1.92-2.17) | 1.92  (1.70-2.17) | 1.62  (1.31-2.02) | 2.32  (1.80-2.98) | <.001 | <.001 | 1.48  (1.40-1.55) | 2.00  (1.83-2.19) | 1.36  (1.11-1.67) | 1.52  (1.29-1.79) | <.001 | <.001 | |
| Household Wealth Level | |  |  |  |  |  |  |  |  |  |  |  | |
| Low | 1.99  (1.86-2.13) | 1.45  (1.28-1.65) | 0.53  (0.39-0.70) | 1.68  (1.37-2.06) | <.001 | <.001 | 1.54  (1.47-1.62) | 2.07  (1.88-2.27) | 1.56  (1.29-1.88) | 1.33  (1.17-1.52) | <.001 | <.001 | |
| Medium | 1.75  (1.62-1.90) | 2.50  (2.04-3.05) | 2.30  (1.54-3.44) | 0.10  (0.05-0.20) | <.001 | <.001 | 1.67  (1.56-1.78) | 2.33  (2.05-2.64) | 1.10  (0.85-1.42) | 0.42  (0.30-0.58) | <.001 | <.001 | |
| High | 2.29  (2.05-2.55) | 5.68  (4.40-7.33) | 3.07  (2.16-4.37) | 8.99  (5.89-13.71) | <.001 | <.001 | 1.40  (1.29-1.53) | 3.07  (2.54-3.72) | 1.00  (.-.)^e^ | 5.15  (3.84-6.90) | <.001 | <.001 | |
| Social Isolation | |  |  |  |  |  |  |  |  |  |  |  | |
| No | 2.55  (2.38-2.74) | 2.11  (1.80-2.46) | 1.16  (0.88-1.53) | 1.10  (0.87-1.40) | <.001 | <.001 | 1.61  (1.53-1.70) | 3.74  (3.30-4.24) | 4.34  (3.17-5.94) | 1.01  (0.80-1.28) | <.001 | <.001 | |
| Yes | 1.56  (1.47-1.66) | 2.02  (1.78-2.30) | 1.27  (0.97-1.65) | 1.51  (1.23-1.86) | <.001 | <.001 | 1.52  (1.45-1.59) | 1.74  (1.60-1.89) | 1.23  (1.04-1.46) | 1.49  (1.31-1.69) | <.001 | <.001 | |

1. Models in overall population were fully adjusted for age, gender, marital status, educational level, rural or urban residence, smoking status, household wealth level, depressive symptoms, social isolation, and body mass index (BMI) category. In subgroup analysis, all other covariates were adjusted for except the stratified variable.
2. ADL: Activity of Daily Living.
3. IADL: Instrumental Activity of Daily Living.
4. Reference: No Functional Dependency.
5. This was omitted as all patients with three IADL dependencies and a high level of household wealth developed multimorbidity in later life.

**eTable 6. Comparison of baseline characteristics between included and excluded participants in CHARLS cohort.**

|  | ADL Sample |  |  | IADL Sample |  |  |
| --- | --- | --- | --- | --- | --- | --- |
| Characteristic | Excluded (n=1632) | Included (n=6773) | P value | Excluded (n=1607) | Included (n=6692) | P value |
| Age | 56.28 (8.90) | 57.66 (8.86) | <0.001 | 56.20 (8.80) | 57.60 (8.82) | <0.001 |
| Gender |  |  | <0.001 |  |  | <0.001 |
| Male | 920 (56.37%) | 3260 (48.13%) |  | 908 (56.50%) | 3230 (48.27%) |  |
| Female | 712 (43.63%) | 3513 (51.87%) |  | 699 (43.50%) | 3462 (51.73%) |  |
| Marital status |  |  | <0.001 |  |  | <0.001 |
| Married | 1181 (72.37%) | 5772 (85.22%) |  | 1163 (72.37%) | 5708 (85.30%) |  |
| Unmarried | 451 (27.63%) | 1001 (14.78%) |  | 444 (27.63%) | 984 (14.70%) |  |
| Education, N (%) |  |  | <0.001 |  |  | <0.001 |
| No formal education/illiterate | 566 (34.68%) | 2986 (44.09%) |  | 557 (34.66%) | 2945 (44.01%) |  |
| Primary school or below | 334 (20.47%) | 1511 (22.31%) |  | 329 (20.47%) | 1500 (22.41%) |  |
| Middle school or above | 719 (44.06%) | 2276 (33.60%) |  | 708 (44.06%) | 2247 (33.58%) |  |
| Area of residence, N (%) | |  | <0.001 |  |  | <0.001 |
| Rural | 899 (55.09%) | 4437 (65.51%) |  | 887 (55.20%) | 4380 (65.45%) |  |
| Urban | 733 (44.91%) | 2336 (34.49%) |  | 720 (44.80%) | 2312 (34.55%) |  |
| Smoking status, N (%) | |  | <0.001 |  |  | <0.001 |
| Never smoker | 909 (55.70%) | 4123 (60.87%) |  | 904 (56.25%) | 4062 (60.70%) |  |
| Former smoker | 63 (3.86%) | 443 (6.54%) |  | 62 (3.86%) | 441 (6.59%) |  |
| Current smoker | 332 (20.34%) | 2207 (32.59%) |  | 331 (20.60%) | 2189 (32.71%) |  |
| BMI, N (%) |  |  | 0.159 |  |  | 0.173 |
| Underweight (<18.5) | 4 (7.55%) | 404 (5.96%) |  | 4 (0.25%) | 401 (5.99%) |  |
| Normal (18.5–24.0) | 29 (54.72%) | 3759 (55.50%) |  | 28 (1.74%) | 3720 (55.59%) |  |
| Overweight (24.0–28.0) | 10 (18.87%) | 1885 (27.83%) |  | 9 (0.56%) | 1862 (27.82%) |  |
| Obese (≥28.0) | 10 (18.87%) | 725 (10.70%) |  | 9 (0.56%) | 709 (10.59%) |  |
| FD Level | ADL | | <0.001 | IADL | | <0.001 |
| 1 | 872 (53.43%) | 2975 (43.92%) |  | 1421 (88.43%) | 5750 (85.92%) |  |
| 2 | 7 (0.43%) | 28 (0.41%) |  | 72 (4.48%) | 533 (7.96%) |  |
| 3 | 6 (0.37%) | 40 (0.59%) |  | 49 (3.05%) | 217 (3.24%) |  |
| ≥4 | 14 (0.86%) | 53 (0.78%) |  | 15 (0.93%) | 109 (1.63%) |  |
| Chronic disease (Following-up) | 733 (44.91%) | 3677 (54.29%) |  | 50 (3.11%) | 83 (1.24%) |  |
| Hypertension | 177 (10.85%) | 838 (12.37%) | 0.083 | 177 (11.01%) | 828 (12.37%) | 0.116 |
| Dyslipidemia | 149 (9.13%) | 566 (8.36%) | 0.259 | 148 (9.21%) | 560 (8.37%) | 0.226 |
| Diabetes | 74 (4.53%) | 312 (4.61%) | 0.979 | 74 (4.60%) | 309 (4.62%) | 1 |
| Heart disease | 101 (6.19%) | 411 (6.07%) | 0.883 | 100 (6.22%) | 401 (5.99%) | 0.771 |
| Stroke | 72 (4.41%) | 296 (4.37%) | 0.959 | 71 (4.42%) | 293 (4.38%) | 0.964 |
| Chronic lung disease | 77 (4.72%) | 292 (4.31%) | 0.506 | 77 (4.79%) | 286 (4.27%) | 0.388 |
| Asthma | 26 (1.59%) | 103 (1.52%) | 0.922 | 26 (1.62%) | 101 (1.51%) | 0.84 |
| Liver disease | 41 (2.51%) | 157 (2.32%) | 0.722 | 41 (2.55%) | 155 (2.32%) | 0.653 |
| Cancer | 27 (1.65%) | 78 (1.15%) | 0.127 | 27 (1.68%) | 76 (1.14%) | 0.098 |
| Digestive disease | 139 (8.52%) | 416 (6.14%) | <0.001 | 135 (8.40%) | 411 (6.14%) | 0.002 |
| Kidney disease | 55 (3.37%) | 188 (2.78%) | 0.23 | 55 (3.42%) | 187 (2.79%) | 0.209 |
| Arthritis | 126 (7.72%) | 480 (7.09%) | 0.664 | 124 (7.72%) | 472 (7.05%) | 0.622 |
| Psychiatric disease | 9 (0.55%) | 56 (0.83%) | 0.341 | 9 (0.56%) | 56 (0.84%) | 0.346 |
| Memory-related disease | 31 (1.90%) | 117 (1.73%) | 0.698 | 29 (1.80%) | 114 (1.70%) | 0.85 |
| Multimorbidity (Following-up), N (%) | 738 (45.22%) | 2995 (44.22%) | 0.482 | 726 (45.18%) | 2956 (44.17%) | 0.484 |

Notes: Continuous variables are presented as mean (standard deviation) and compared using Student’s t-test. Categorical variables are presented as number (percentage) and compared using chi-square test.FD, functional dependency; ADL, Activities of Daily Living; IADL, Instrumental Activities of Daily Living.

eTable 7. Comparison of baseline characteristics between included and excluded participants in SHARE cohort.

|  | ADL/IADL Sample |  |  |
| --- | --- | --- | --- |
| Characteristic | Excluded (n=10062) | Included (n=6574) | P value |
| Age | 64.14 (8.63) | 61.86 (8.63) | <0.001 |
| Gender |  |  | 0.212 |
| Male | 4341 (43.14%) | 2771 (42.15%) |  |
| Female | 5721 (56.86%) | 3803 (57.85%) |  |
| Marital status |  |  | <0.001 |
| Married | 4078 (40.53%) | 4135 (62.90%) |  |
| Unmarried | 1159 (11.52%) | 2439 (37.10%) |  |
| Education, N (%) |  |  | 0.318 |
| No formal education/illiterate | 4018 (39.93%) | 2978 (45.30%) |  |
| Primary school or below | 2364 (23.49%) | 1683 (25.60%) |  |
| Middle school or above | 0 (0.00%) | 0 (0.00%) |  |
| Area of residence, N (%) |  |  | 0.055 |
| Rural | 1281 (12.73%) | 1481 (22.53%) |  |
| Urban | 2124 (21.11%) | 2236 (34.01%) |  |
| Town | 1822 (34.86%) | 2513 (40.34%) |  |
| Smoking status,N (%) |  |  | 0.328 |
| Never smoker | 2726 (27.09%) | 3700 (56.28%) |  |
| Former smoker | 1192 (11.85%) | 1513 (23.01%) |  |
| Current smoker | 1040 (10.34%) | 1361 (20.70%) |  |
| FD Level |  |  | 0.034 |
| 1 | 118 (1.17%) | 63 (0.96%) |  |
| 2 | 4030 (40.05%) | 2705 (41.15%) |  |
| 3 | 3904 (38.80%) | 2709 (41.21%) |  |
| ≥4 | 1458 (14.49%) | 1097 (16.69%) |  |
| Chronic disease (Following-up) |  |  |  |
| Hypertension | 1854 (18.43%) | 2161 (32.87%) | <0.001 |
| Hypercholesterolemia | 582 (5.78%) | 1057 (16.08%) | <0.001 |
| Diabetes | 289 (2.87%) | 429 (6.53%) | <0.001 |
| Heart disease | 310 (3.08%) | 512 (7.79%) | <0.001 |
| Stroke | 101 (1.00%) | 145 (2.21%) | <0.001 |
| Chronic lung disease | 163 (1.62%) | 267 (4.06%) | <0.001 |
| Hip fracture | 35 (0.35%) | 85 (1.29%) | <0.001 |
| Other fracture | 112 (1.11%) | 210 (3.19%) | <0.001 |
| Arthritis | 229 (2.28%) | 475 (7.23%) | <0.001 |
| Cataracts | 178 (1.77%) | 352 (5.35%) | <0.001 |
| Parkinson disease | 41 (0.41%) | 28 (0.43%) | 0.954 |
| Osteoporosis | 809 (8.04%) | 956 (14.54%) | <0.001 |
| Alzheimer's disease | 108 (1.07%) | 95 (1.45%) | 0.039 |
| Cancer | 209 (2.08%) | 228 (3.47%) | <0.001 |
| Digestive disease | 49 (0.49%) | 149 (2.27%) | <0.001 |
| Multimorbidity (Following-up),N (%) | 0 (0.00%) | 2336 (35.53%) | <0.001 |

Notes: Continuous variables are presented as mean (standard deviation) and compared using Student’s t-test. Categorical variables are presented as number (percentage) and compared using chi-square test.FD, functional dependency; ADL, Activities of Daily Living; IADL, Instrumental Activities of Daily Living.

**eFigure 1. Study flowchart of participant selection**

**
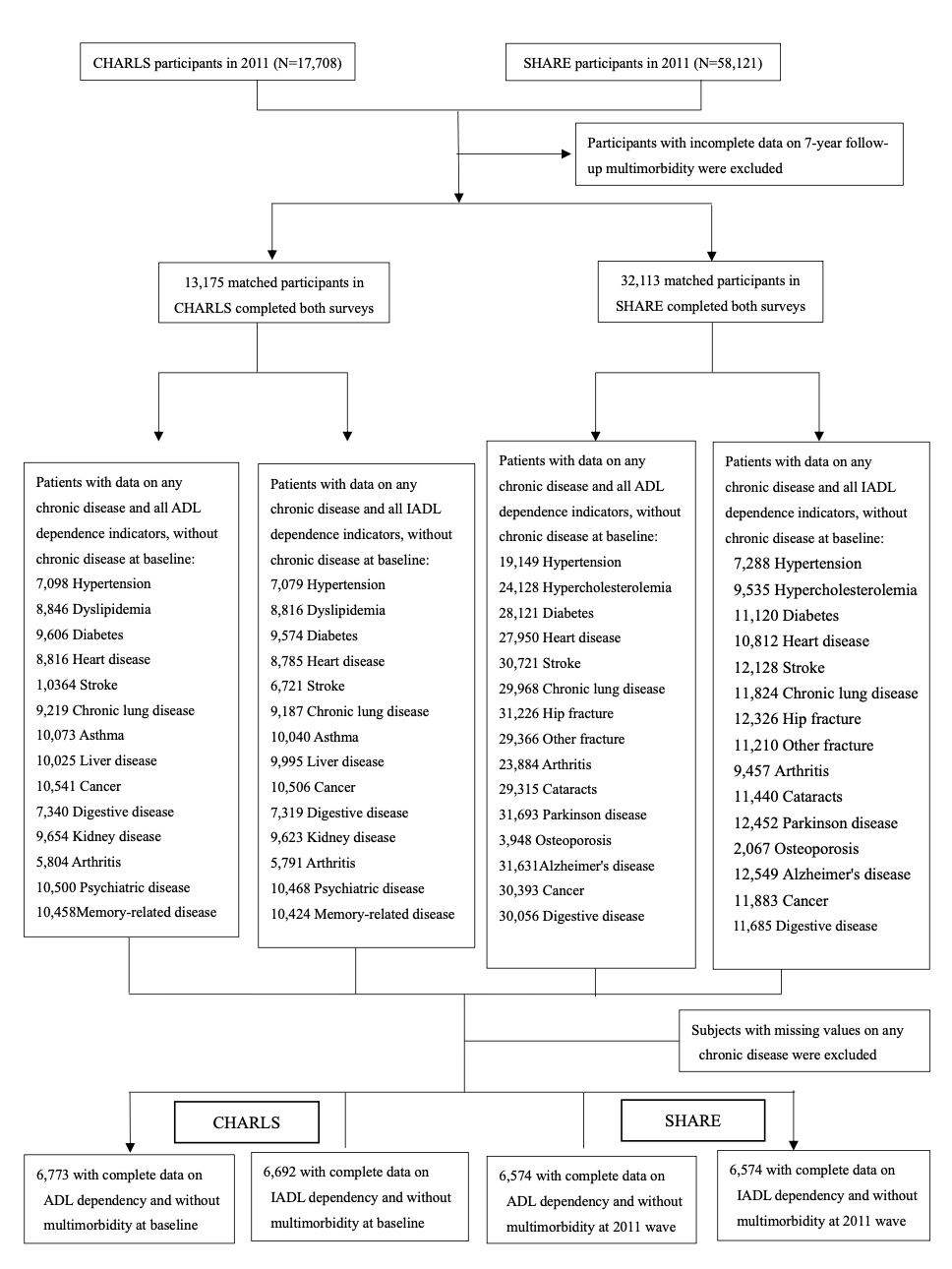
**

**eFigure 2. Association between individual functional dependency indicator and incident multimorbidity**

**
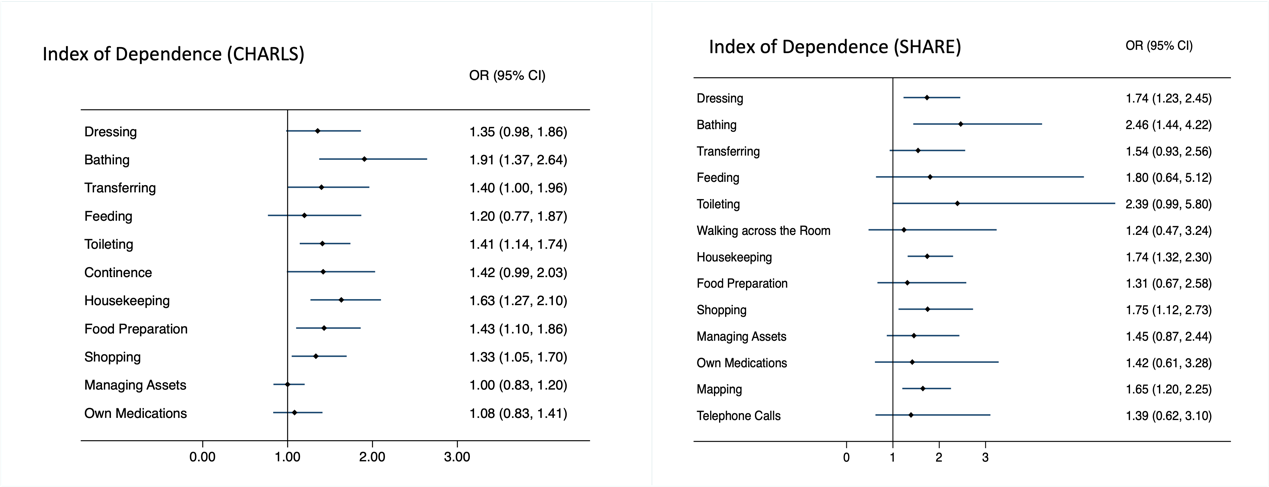
**

Notes: All Models were fully adjusted for age, gender, marital status, educational level, rural or urban residence, smoking status, household wealth level, depressive symptoms, social isolation, and body mass index (BMI) category.

**eFigure 3. Combined associations between level of functional dependency and follow-up incident multimorbidity**

**
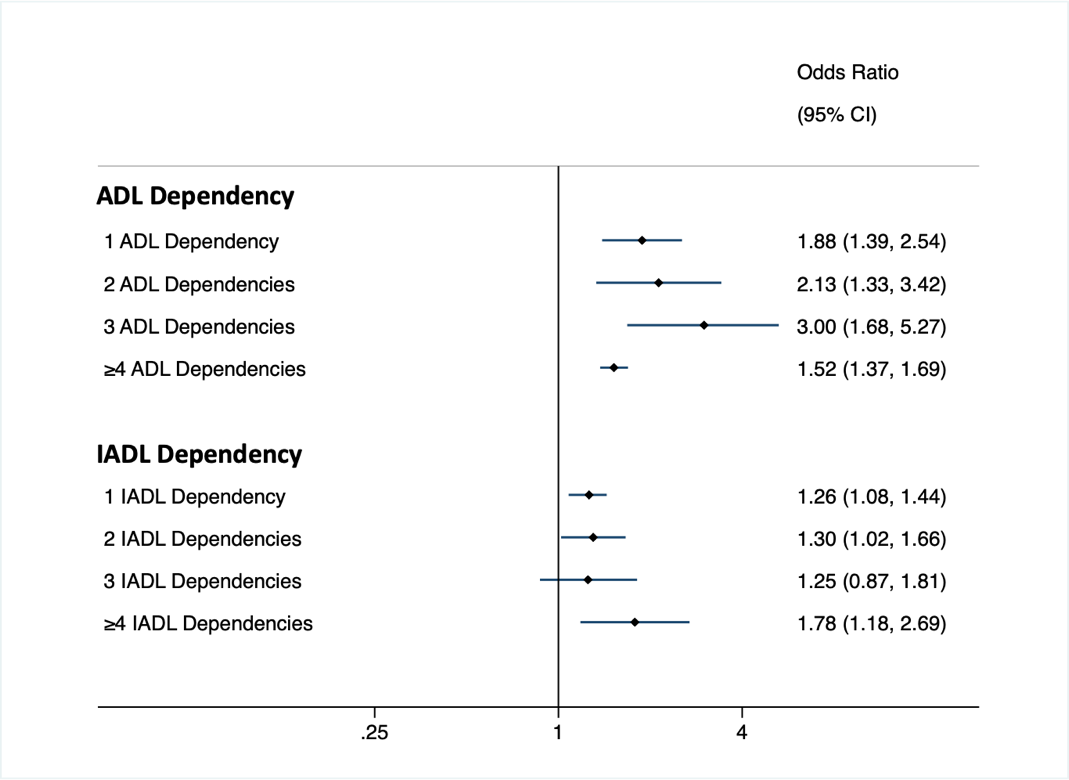
**

**eReference**

1. Deaton A, Zaidi S. *Guidelines for constructing consumption aggregates for welfare analysis*. vol 135. World Bank Publications; 2002.

2. Yu B, Steptoe A, Niu K, Ku P-W, Chen L-J. Prospective associations of social isolation and loneliness with poor sleep quality in older adults. *Quality of Life Research*. 2018/03/01 2018;27(3):683-691. doi:10.1007/s11136-017-1752-9

3. Gale CR, Westbury L, Cooper C. Social isolation and loneliness as risk factors for the progression of frailty: the English Longitudinal Study of Ageing. *Age Ageing*. May 1 2018;47(3):392-397. doi:10.1093/ageing/afx188

4. Litwin H, Sapir EV. Perceived Income Adequacy Among Older Adults in 12 Countries: Findings From the Survey of Health, Ageing, and Retirement in Europe. *The Gerontologist*. 2009;49(3):397-406. doi:10.1093/geront/gnp036

5. Wu Y, Ma G, Hu Y, et al. The current prevalence status of body overweight and obesity in China: data from the China National Nutrition and Health Survey. *Zhonghua yu fang yi xue za zhi [Chinese journal of preventive medicine]*. 2005;39(5):316-320.

6. WHO C. Obesity: preventing and managing the global epidemic. *World Health Organ Tech Rep Ser*. 2000;894(i-xii):1-253.

7. Andresen EM, Malmgren JA, Carter WB, Patrick DL. Screening for depression in well older adults: evaluation of a short form of the CES-D (Center for Epidemiologic Studies Depression Scale). *Am J Prev Med*. Mar-Apr 1994;10(2):77-84.

8. Gallagher D, Savva GM, Kenny R, Lawlor BA. What predicts persistent depression in older adults across Europe? Utility of clinical and neuropsychological predictors from the SHARE study. *J Affect Disord*. May 2013;147(1-3):192-7. doi:10.1016/j.jad.2012.10.037
